# A MAP-SNARE interaction links cortical microtubules to the exocytic fusion machinery during auxin-dependent growth remodeling

**DOI:** 10.1126/sciadv.aef5793

**Published:** 2026-09-02

**Authors:** Jia Deng, Jing Qin, Panpan Wang, Xiaojing Zhao, Xinyuan Zhang, Jingyan Fu, Tonglin Mao, Xiangfeng Wang

**Affiliations:** ^1^ State Key Laboratory of Plant Environmental Resilience, Department of Plant Sciences, College of Biological Sciences, China Agricultural University, Beijing 100193, China.; ^2^ State Key Laboratory of Animal Biotech Breeding, College of Biological Sciences, China Agricultural University, Beijing 100193, China.

## Abstract

Auxin exerts diverse physiological effects through local maxima and tissue-scale gradients established by directional transport, which depends on the plasma membrane (PM) distribution and vesicular trafficking of auxin transporters. Cortical microtubules are positioned beneath the PM and have been implicated in trafficking events. However, how microtubules interface with terminal vesicle trafficking machinery at the PM remains unclear. Here, using auxin-dependent apical hook opening in Arabidopsis, we identify a molecular bridge linking cortical microtubules to PM-associated SNARE machinery. The microtubule-associated protein WDL4 interacts with the PM Qa-SNARE SYP121 and connects it to cortical microtubules through separate SNARE-binding and microtubule-binding domains. This coupling enhances the interaction between SYP121 and its R-SNARE partner VAMP721. Consistently, PM-localized VAMP721 signals were reduced in *wdl4* mutants. Disruption of WDL4 or SYP121 impaired PM accumulation and recycling of the auxin efflux carrier PIN1 and attenuated apical hook opening. Our findings elucidate a mechanism by which a microtubule-associated factor modulates SNARE-mediated auxin transporter trafficking during auxin-dependent growth remodeling.

## INTRODUCTION

The phytohormone auxin is a major regulator of plant growth and development, and mediates plant adaptation to environmental signals (*1*, *2*). A key feature of auxin is its active directional intercellular transport, which generates local auxin maxima and concentration gradients to instruct specific cellular behavior and developmental outputs (*3*). During soil emergence, dicotyledon seedlings form a unique apical hook structure to protect the shoot apical meristem and cotyledons from potential mechanical damage (*4*). Apical hook development in Arabidopsis proceeds through three phases: formation, maintenance, and opening, which provides a classical model of auxin-mediated differential cell elongation (*5*–*7*). Auxin accumulation at the inner (concave) side of the hypocotyl restricts the cell elongation, facilitating hook formation (*6*, *7*). Decrease in this local auxin maximum alleviates the inhibition of cell elongation and ultimately leads to hook opening (*6*, *8*).

The establishment and regulation of precise auxin patterns primarily depend on auxin transporters asymmetrically distributed at the plasma membrane (PM), such as influx carriers AUX/LIKE-AUX (LAX) and efflux carriers PIN-FORMED (PIN) proteins (*6*, *9*–*11*). Crucially, the polarity and abundance of auxin transporters at the PM are regulated by highly dynamic vesicular trafficking processes. While the endocytosis and endosomal sorting of auxin transporters have been extensively studied, efficient PM delivery of these transporters through either de novo secretion or recycling ultimately requires a terminal vesicle-PM fusion step (*11*–*19*). This fusion event is mediated by SNARE (soluble N-ethylmaleimide-sensitive factor attachment protein receptor) protein complexes, such as those formed by Qa-SNARE SYP121 (Syntaxin of Plants 121) together with its cognate R-SNAREs VAMP721/722 (vesicle-associated membrane protein 721/722) (*20*–*24*). Consistent with the essential role of this machinery in the trafficking of auxin transporters, the *vamp721 vamp722* double mutant exhibited aberrant auxin distribution in roots and severe development defects (*25*). However, how SNARE complex assembly is regulated to support the rapid remodeling of transporter distribution and auxin gradients in growth and development remains to be elucidated.

The microtubule cytoskeleton is essential for plant growth, development, and environmental adaptation. Cortical microtubules are positioned immediately beneath the PM and are traditionally viewed as tracks guiding the orientation of cellulose microfibril deposition in the cell wall, thereby regulating cell expansion (*13*, *26*–*32*). Emerging evidence also highlights the role of microtubule-based intracellular transport in various physiological responses (*33*, *34*). For example, the microtubule plus-end-binding protein END BINDING1b (EB1b) interacts with SYP121 to facilitate the trafficking and PM localization of the K^+^ channel KAT1 during light-induced stomatal opening (*34*). Previous studies suggest that microtubules can influence auxin patterning through trafficking-related mechanisms. Treatment with microtubule-disrupting drugs has been reported to affect PINs trafficking (*35*, *36*). The microtubule-associated protein (MAP) CYTOPLASMIC LINKER ASSOCIATED PROTEIN (CLASP) mediates an association of sorting nexin 1 (SNX1) endosomes with microtubules, facilitating the endocytic recycling of PIN2 and restricting its degradation during root growth (*36*). These studies suggest a role of CLASP-microtubule module in endosomal sorting and recycling. However, the regulatory mechanisms of the terminal fusion machinery during auxin-dependent growth remodeling, particularly the roles of MAPs and microtubules, remain largely unknown.

In this study, we identify a molecular link between the cortical microtubules and the PM SNARE machinery. We show that the PM Qa-SNARE protein SYP121 positively regulates the apical hook opening in Arabidopsis, and demonstrate that MAP WAVE-DAMPENED-LIKE4 (WDL4) physically and genetically interacts with SYP121. Through separate SNARE-interaction and microtubule-binding domains, WDL4 links SYP121 to cortical microtubules. Moreover, we show that WDL4 enhances the interaction between SYP121 and its cognate R-SNARE VAMP721. Together, these results support a regulatory mode in which a MAP-SNARE interaction facilitates SNARE pairing, thereby promoting the remodeling of auxin transporter distribution during auxin-dependent growth and development.

## RESULTS

### SNARE protein SYP121 physically interacts with WDL4 and positively regulates apical hook opening

**Fig. 1.**
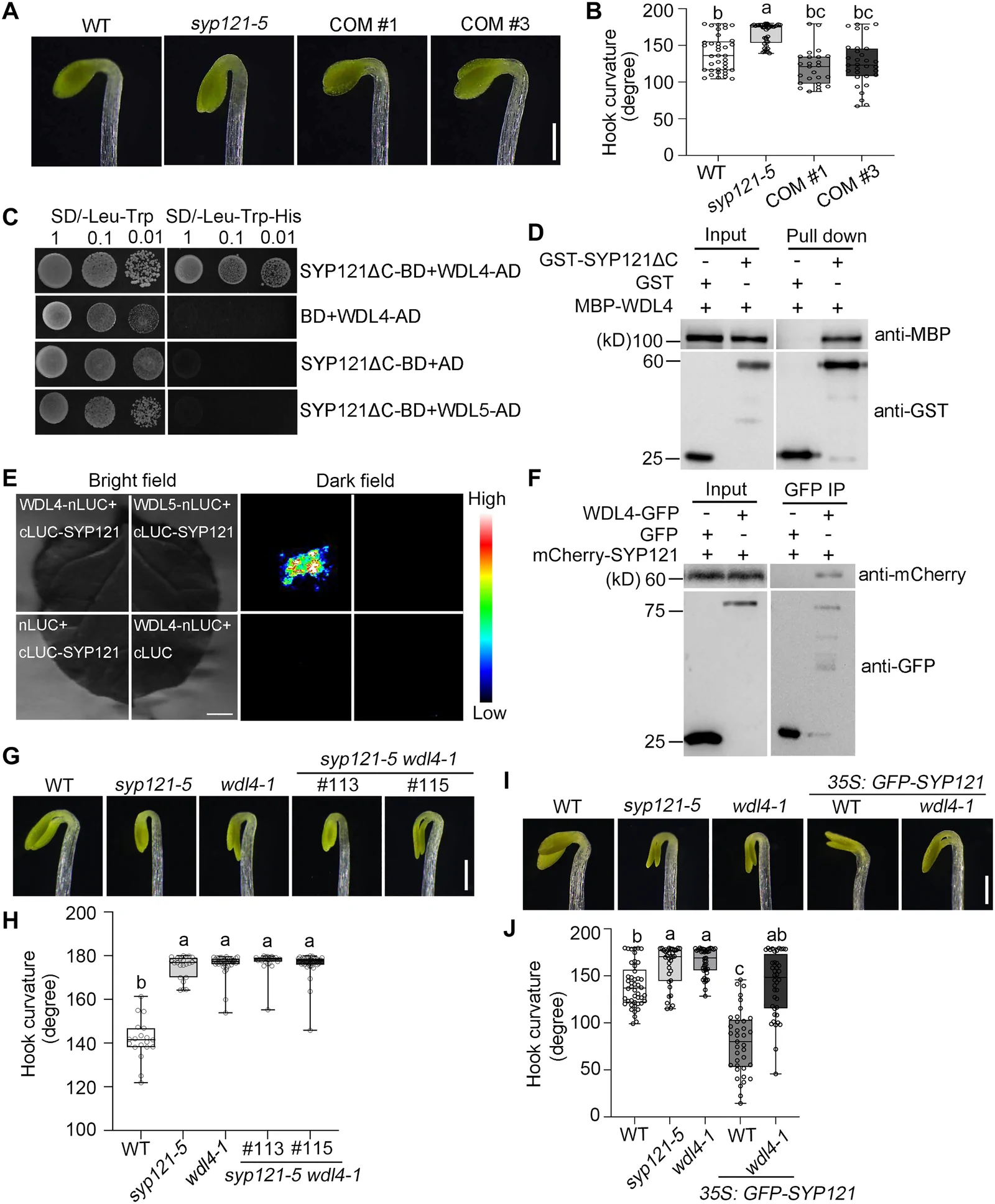
SYP121 physically interacts with WDL4 and regulates apical hook opening. (**A**) and (**B**) Representative images and quantitative analysis of the hook curvature in 3-day-old etiolated seedlings as indicated. Data are shown as box plots with individual data points (*n* ≥ 25 seedlings). Scale bar, 0.5 mm. (**C**) Yeast two-hybrid assay showing the interaction between the cytosolic domain of SYP121 (SYP121ΔC) and WDL4, but not WDL5. Empty vectors were used as negative controls. 5 mM 3-AT was used to inhibit autoactivation. (**D**) In vitro pull-down assay showing the interaction between MBP-WDL4 and GST-SYP121ΔC. GST was used as a negative control. (**E**) Firefly luciferase complementation imaging (LCI) assay in *N. benthamiana* leaves. Signals were detected with the co-transformation of *WDL4-nLUC* and *cLUC-SYP121*. nLUC and cLUC indicate the N-terminal and C-terminal fragments of firefly Luciferase. Empty vectors and WDL5-nLUC were used as negative controls. Scale bar, 1 cm. (**F**) Co-IP assay showing the interaction between WDL4-GFP and mCherry-SYP121 in vivo. GFP was used as a negative control. (**G**) and (**H**) Representative images and quantification of the hook curvature in 3-day-old etiolated seedlings as indicated. Scale bar, 0.5 mm. Data are shown as box plots with individual data points (*n* ≥ 19 seedlings). (**I**) and (**J**) Representative images and quantification of the hook curvature in 3-day-old etiolated seedlings as indicated. Scale bar, 0.5 mm. Data are shown as box plots with individual data points (*n* ≥ 34 seedlings). For (A), (G) and (I), seedlings were germinated and grown vertically in the dark for 72 h before imaging. Statistical significance in (B), (H), and (J) was determined by Kruskal-Wallis test followed by Dunn’s multiple comparisons test with Bonferroni-adjusted *P* values. Different letters indicate significant differences at *P* < 0.05. Experiments were repeated three times independently with similar results.

To investigate the role and regulatory mechanisms of SNARE-mediated trafficking in auxin-dependent growth remodeling, we focused on the Qa-SNARE SYP121, which is a major component of the plant PM delivery machinery (*37*). We used apical hook development in Arabidopsis as a model, and obtained the T-DNA insertion mutant *syp121–5* (*24*) for hook phenotype analysis. Compared to wild-type (WT) seedlings, hook curvature in *syp121–5* mutant was more exaggerated when grown in the dark for 3 days, indicating that hook opening was delayed (Fig. 1, A and B, fig. S1). This phenotype was complemented when *SYP121pro: GFP-SYP121* was introduced into *syp121–5* mutant (Fig. 1, A and B, figs. S1 and S2). Meanwhile, we generated another mutant allele in *SYP121* (named as *syp121–7*) using CRISPR/Cas9 (*38*) and its complementation lines to provide further support for this phenotype (fig. S3). These results demonstrate that SYP121 positively regulates apical hook opening in Arabidopsis seedlings.

Previous proteomic study by immunoprecipitation (IP)-mass spectrometric (MS) indicated the possible link between SYP121 and a MAP WDL4, which has been shown to be a positive regulator of apical hook opening (*39*). To validate the interaction between SYP121 and WDL4, we first performed the yeast two-hybrid assay using the cytosolic fragment of SYP121 (SYP121ΔC) as previously reported (*40*). SYP121ΔC showed specific interaction with WDL4, but no interaction with WDL5, a close homolog of WDL4 (Fig. 1C). Next, we performed in vitro pull-down assay using proteins expressed and purified from *Escherichia coli*. MBP-WDL4 fusion protein was pulled down by GST-SYP121ΔC, but not by GST protein alone (Fig. 1D). We further confirmed the in vivo interaction between SYP121 and WDL4 using luciferase complementation imaging (LCI). Co-expression of WDL4 with full-length SYP121 in *Nicotiana benthamiana* leaves resulted in strong luciferase (Luc) activity. In contrast, no Luc activity was detected when WDL5 was co-expressed with SYP121, serving as a negative control (Fig. 1E). Moreover, we performed a co-immunoprecipitation (Co-IP) assay using *N. benthamiana* leaves transiently expressing *mCherry-SYP121* together with *WDL4-GFP* or *GFP* alone. mCherry-SYP121 coimmunoprecipitated with WDL4-GFP, but not GFP negative control (Fig. 1F). Together, these results suggest that SYP121 physically interacts with WDL4 in vitro and in vivo.

The physical interaction between SYP121 and WDL4, together with their similar phenotypes with delayed hook opening, prompted us to examine their genetic relationship. We generated *syp121–5 wdl4–1* double mutant by crossing. Phenotype analysis showed that the *syp121–5 wdl4–1* double mutant exhibited a similar hook-opening defect comparable to either single mutant (Fig. 1, G and H). We further obtained the *SYP121* overexpressing line carrying *35S: GFP-SYP121* transgene to examine the effect of increasing SYP121 dosage. *SYP121* overexpression accelerated hook opening in the WT background, but the effect was much weaker in the *wdl4–1* background (Fig. 1, I and J, fig. S2). These results indicate that WDL4 contributes to the full effect of *SYP121* overexpression in promoting hook opening. Together, these physical interaction and genetic results support a functional connection between SYP121 and WDL4 during hook opening.

### WDL4 connects SYP121 with cortical microtubules through distinct domains

**Fig. 2.**
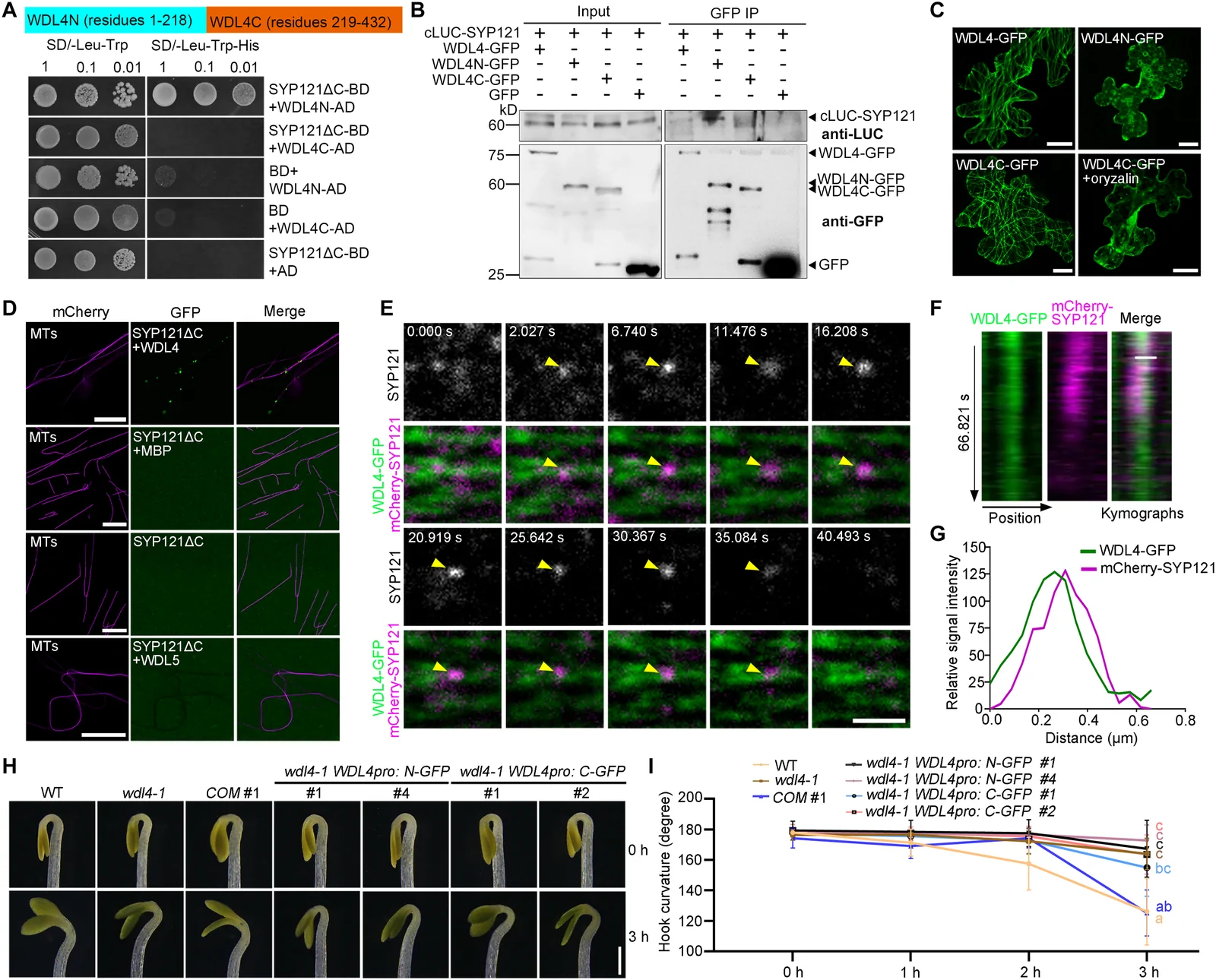
WDL4 connects SYP121 with cortical microtubules through distinct domains. (**A**) Yeast two-hybrid assay showing that WDL4N (residues 1–218) interacts with the cytosolic domain of SYP121 (SYP121ΔC), whereas WDL4C (residues 219–432) does not. 5 mM 3-AT was used to inhibit autoactivation. (**B**) Co-IP analysis showing the interaction between SYP121 and WDL4 truncations in vivo. WDL4-GFP and GFP were used as positive and negative controls respectively. (**C**) Ballistics-mediated transient expression of WDL4 truncations in Arabidopsis (Col-0) leaf epidermal cells. The localization of WDL4C-GFP was observed following DMSO or oryzalin treatment. Scale bars, 20 μm. (**D**) Representative images of microtubules incubated with His-SYP121ΔC-GFP using in vitro system. MBP-WDL4, MBP and MBP-WDL5 were added to the system respectively. Magenta fluorescent signals indicate rhodamine-labeled microtubules, and green signals indicate His-SYP121ΔC-GFP fusion protein. Scale bars, 10 μm. (**E**) VA-TIRF microscopy imaging showed mCherry-SYP121 (magenta) associates with WDL4-labeled filamentous structures (green) for a certain duration in stable transgenic Arabidopsis plants. Both *WDL4-GFP* and *mCherry-SYP121* were driven by their native promoters. Arrowheads indicate SYP121 punctate signal. Scale bar, 1 μm. (**F**) Representative kymograph showing the association of SYP121 (magenta) with WDL4 (green) as shown in (E). (**G**) Plot of the line scan drawn in (F) showing the association between mCherry-SYP121 and WDL4-GFP. (**H**) and (**I**) Representative images and quantification of apical hook curvature in different seedlings as indicated. Seedlings were germinated and grown vertically in the dark for 42 h (0 h), followed by short-term white light treatment as indicated. Scale bar, 0.5 mm. Values represent mean ± SD (*n* ≥ 8 seedlings). Statistical significance was determined by Kruskal-Wallis test followed by Dunn’s multiple comparisons test with Bonferroni-adjusted *P* values. Different letters indicate significant differences at *P* < 0.05. Experiments were repeated three times independently with similar results.

To understand how WDL4 could affect the function of SYP121, we first determined the protein domain of WDL4 mediating its interaction with SYP121. WDL4 is predicted to contain TPX2 and two coiled-coil domains at the C terminal (*41*, *42*). We generated two truncation variants, WDL4N (residues 1–218) and WDL4C (residues 219–432), and performed the yeast two-hybrid assay with SYP121ΔC. As shown in Fig. 2A, SYP121ΔC interacted with WDL4N but not WDL4C. Furthermore, the in vivo interaction between WDL4N and full length SYP121 was validated by the Co-IP assay in *N. benthamiana* leaves (Fig. 2B). Next, we performed ballistics-mediated transient expression of *35S: WDL4N-GFP* and *35S: WDL4C-GFP* in Arabidopsis leaf epidermal cells. The localization analysis indicated that the WDL4N-GFP signal primarily localized to the cytoplasm, whereas the WDL4C-GFP signal exhibited filamentous structures that were disrupted with the application of the microtubule-disrupting drug oryzalin (Fig. 2C). Thus, WDL4N and WDL4C fragments mediated the interaction with SYP121 and association with CMTs respectively. This functional segmentation in WDL4 suggested that WDL4 could serve as a potential linker between SYP121 and the cortical microtubules.

To test this hypothesis, we performed an in vitro assay by incubating paclitaxel-stabilized microtubules (pre-polymerized from rhodamine-labeled tubulin) with MBP-WDL4 and His-SYP121ΔC-GFP fusion proteins that were purified from *E. coli*. Confocal microscopy showed that SYP121ΔC-GFP punctate signals located on the microtubules when MBP-WDL4 was introduced into the system, whereas this phenomenon was not observed with the addition of MBP or MBP-WDL5 (Fig. 2D). We further examined the association of SYP121 and WDL4-labeled cortical microtubules in vivo. We generated a transgenic line expressing both *WDL4-GFP* and *mCherry-SYP121* driven by their native promoters (Fig. 2E), as well as a transgenic line overexpressing both *GFP-SYP121* and *mCherry-WDL4* (fig. S4). The seedlings were subjected to dual-channel time-lapse imaging by variable angle-total internal reflection fluorescence microscopy (VA-TIRFM). Punctate signals of both mCherry-SYP121 and GFP-SYP121 on the PM were observed, while WDL4-GFP and mCherry-WDL4 signals were visualized along the filamentous cortical microtubules that are closely adjacent to the PM (Fig. 2E, fig. S4). Time-lapse imaging with subsequent kymograph analysis showed that certain SYP121 punctate signals coexisted with the WDL4-labeled filamentous structures and remained stable for a certain duration (Fig. 2, F and G, fig. S4, and movie S1).

Notably, single fragment of both WDL4N and WDL4C failed to rescue the delayed hook opening phenotype observed in the *wdl4–1* mutants in the light-triggered rapid hook opening system (*8*, *43*, *44*) (Fig. 2, H and I), indicating that both the interaction with SYP121 and association with cortical microtubules are necessary for the functioning of WDL4 in regulating hook opening. Taken together, these findings suggested a connection between SYP121 and WDL4-labeled cortical microtubules, supporting their functional interplay in the regulation of hook opening.

### SYP121-WDL4 module regulates PIN1 distribution during apical hook opening

**Fig. 3.**
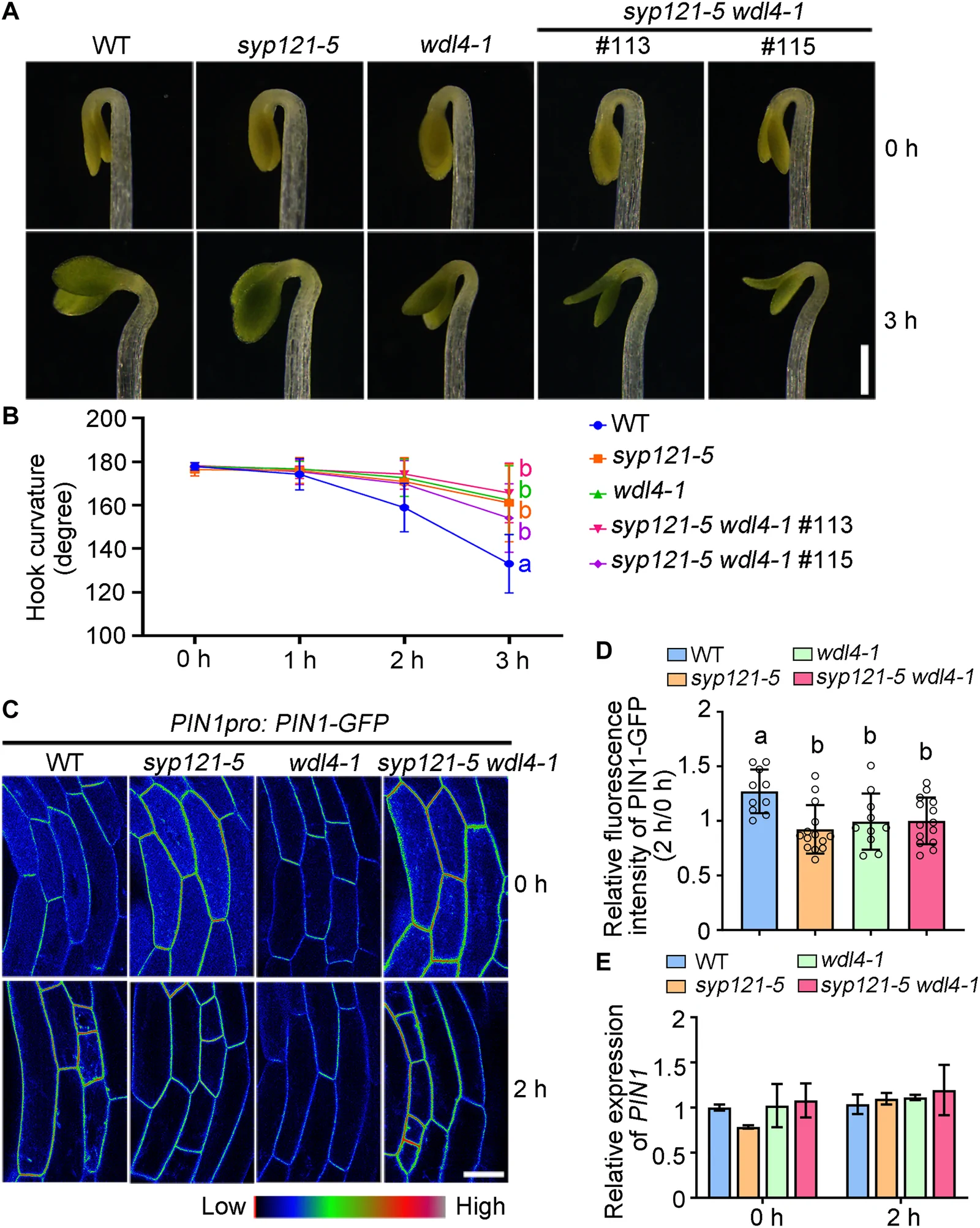
SYP121-WDL4 module regulates PIN1 distribution during apical hook opening. **A**) and (**B**) Light-triggered rapid hook opening process in WT, *syp121–5*, *wdl4–1*, and two independent *syp121–5 wdl4–1* lines. Seedlings were germinated and grown vertically in the dark for 42 h (0 h), followed by short-term white light treatment as indicated. Representative images were taken, and hook curvature was quantified at different time points as labeled. Values in (B) represent mean ± SD (n ≥ 9 seedlings). Statistical significance was determined by Kruskal-Wallis test followed by Dunn’s multiple comparisons test with Bonferroni-adjusted *P* values. Different letters indicate significant differences at *P* < 0.05. Experiments were repeated three times independently with similar results. Scale bar, 0.5 mm. (**C**) Representative images of PIN1-GFP subcellular distribution in hook epidermal cells in WT, *syp121–5*, *wdl4–1*, and *syp121–5 wdl4–1* seedlings expressing *PIN1pro: PIN1-GFP*. PIN1-GFP signals were shown as pseudocolors (red-green-blue palette). Seedlings were germinated and grown in the dark for 42 h (0 h), followed by white light illumination for 2 h. Scale bar, 20 μm. (**D**) Light-triggered induction of PM-localized PIN1-GFP in WT, *syp121–5*, *wdl4–1*, and *syp121–5 wdl4–1* seedlings. The relative fluorescence intensity before (0 h) and after (2 h) white light treatment was quantified and the ratio (2 h/0 h) was calculated. Values represent mean ± SD (n ≥ 10 seedlings). Significant differences are determined by one-way ANOVA followed by Tukey’s multiple comparisons test. Different letters indicate significant differences at *P* < 0.05. Experiments were repeated three times independently with similar results. (**E**) Relative expression of *PIN1* in the apical hook of the seedlings used in (C). One to two millimeters of the apical hook regions from seedlings were used for RNA extraction. Values represent mean ± SD for three independent experiments.

We further confirmed the functional interaction between SYP121 and WDL4 using the light-triggered rapid hook opening system. Seedlings were initially grown in the dark for 42 h after germination, at which stage all genotypes displayed closed hooks uniformly, as shown in Fig. 3A. Following white light illumination for 1–3 h, apical hook opening was observed in WT seedlings, while this induction was significantly impaired in *syp121–5* and *wdl4–1* mutants. The *syp121–5 wdl4–1* double mutant exhibited a similar phenotype comparable to either single mutant (Fig. 3, A and B). These results are consistent with the delayed hook-opening phenotypes observed in the dark (Fig. 1).

Apical hook opening depends on differential cell growth between the concave and convex sides of the hook (*6*). To clarify the cellular basis of the delayed hook-opening phenotype, we quantified epidermal cell length on both sides of the apical hook at the stage when the phenotype was observed. Epidermal cells on the concave side were shorter in *syp121–5*, *wdl4–1*, and *syp121–5 wdl4–1* mutant seedlings than in WT seedlings, whereas enhanced elongation on the convex side was not observed (fig. S5). These results indicate that reduced concave-side cell elongation is associated with the delayed hook-opening phenotype in these mutants.

Auxin gradient established by polar auxin transport is closely linked to differential cell growth during apical hook opening. A decrease in the local auxin maximum on the concave side relieves growth inhibition and promotes hook opening (*7*). Recent studies have shown that WDL4 contributes to auxin distribution by modulating the abundance of auxin efflux carriers at the PM (*44*, *45*). Taking into account the genetic evidence presented in Fig. 1 and Fig. 3, these results prompted us to test whether SYP121 and WDL4 are functionally connected in regulating the abundance of auxin efflux carriers. To validate this hypothesis, we first crossed the transgenic line expressing auxin response reporter *DR5: GFP* with the *syp121–5* mutant (*46*). Compared to WT seedlings, the *syp121–5* mutant displayed stronger DR5: GFP signals in the concave side of the apical hook, which is similar to *wdl4–1* mutant (fig. S6) (*44*). Next, we examined the cellular localization of PIN1-GFP in the hook epidermal cells of WT, *syp121–5*, *wdl4–1*, and *syp121–5 wdl4–1* seedlings using the light-induced hook opening system. We quantified the change of PIN1-GFP intensity induced by white light exposure. White light exposure induced an increased PIN1-GFP signal at the PM in epidermal cells of WT hook regions, but this effect was not observed in *wdl4–1* mutant, *syp121–5* mutant and *syp121–5 wdl4–1* double mutant (Fig. 3, C and D). In addition, RT-qPCR analysis showed that the expression level of *PIN1* was consistent in the hook region of these seedlings, whether subjected to light exposure or not, indicating that the difference of PIN1-GFP intensity is not due to variations in *PIN1* transcript level (Fig. 3E). These results further supported the functional connection between SYP121 and WDL4 in regulating PINs distribution and auxin gradient during apical hook opening.

To further clarify which step of PIN1 trafficking was affected, we performed brefeldin A (BFA) treatment and washout assays (*44*). For BFA treatment, etiolated seedlings grown for 42 h were illuminated with white light and pre-treated with cycloheximide (CHX) for 1 h to inhibit de novo protein biosynthesis, followed by CHX + BFA treatment for 30 min. Endocytosed PIN1-GFP was trapped in the BFA bodies. We observed no significant difference in the number or relative size of BFA bodies among WT, *syp121–5*, *wdl4–1*, and *syp121–5 wdl4–1* seedlings, indicating that PIN1-GFP endocytosis was not detectably affected in these mutants under the tested conditions (fig. S7, A to C). For the BFA washout assays, seedlings were pre-treated with CHX for 30 min, followed by CHX + BFA treatment for 1.5 h and BFA washout while maintaining CHX. After BFA washout, PIN1-GFP-labeled BFA bodies were less efficiently reduced in number and relative size in the mutants than in WT seedlings (fig. S7, D to F), indicating impaired PIN1 recycling back to the PM. Together, these results suggest that the WDL4-SYP121 module is required for efficient recycling of PIN1 back to the PM.

To further test whether the delayed hook-opening phenotype is functionally linked to auxin transport, we used the auxin efflux inhibitor NPA to pharmacologically perturb auxin transport during light-induced hook opening. The *syp121–5*, *wdl4–1*, and *syp121–5 wdl4–1* seedlings showed lower relative hook opening under NPA treatment than WT seedlings (fig. S8), indicating enhanced sensitivity to auxin efflux inhibition. Together with the PIN1 recycling defect, these results support a functional link between the WDL4-SYP121 module and auxin transport-dependent hook opening.

### WDL4 interacts with the N terminus of SYP121

**Fig. 4.**
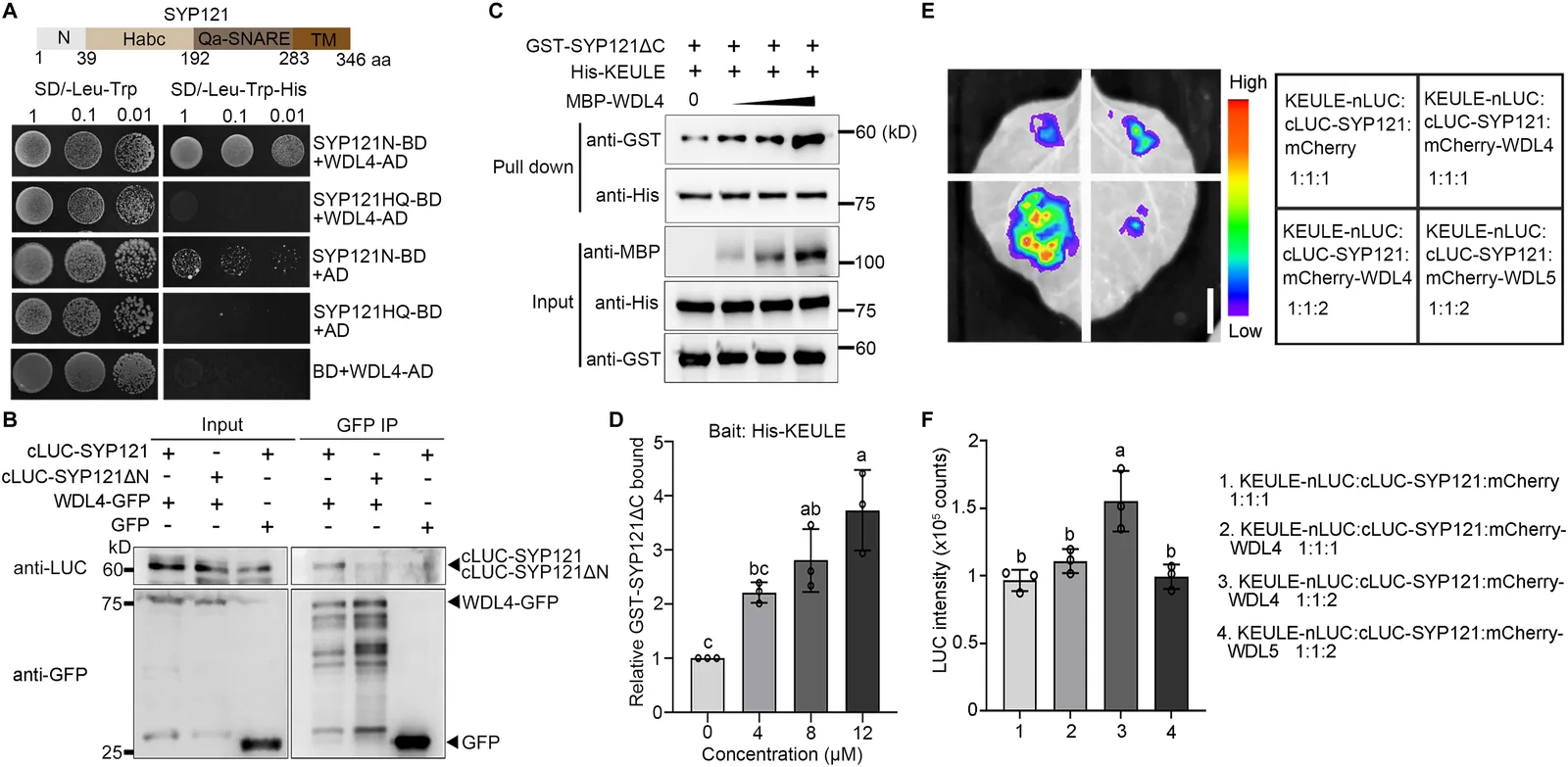
WDL4 interacts with the N-terminal of SYP121 and facilitates the interaction between SYP121 and KEULE. (**A**) Yeast two-hybrid assays showing the interaction between WDL4 and SYP121 truncations. SYP121 truncations were generated according to four distinct domains of SYP121, as shown in the diagram. N, unstructured N-terminal domain (residues 1–39); H, Habc domain (residues 40–192); Q, H3 coiled-coil domain (which includes the Qa-SNARE motif) (residues 193–283); TM, C-terminal membrane anchor (residues 284–346). Empty vectors were used as negative controls. 5 mM 3-AT was used to inhibit autoactivation. (**B**) In vivo Co-IP assay indicated that the interaction between WDL4 and SYP121 depends on the N-terminal of SYP121. WDL4-GFP and GFP were immunoprecipitated and the coimmunoprecipitated proteins were detected using an anti-LUC antibody. GFP was used as a negative control. (**C**) Pull-down assay showed that WDL4 enhances the interaction between SYP121 and KEULE in vitro. Affinity-purified GST-SYP121ΔC and His-KEULE were incubated with gradient concentrations of MBP-WDL4. Increasing amount of GST-SYP121ΔC was pulled down by His- KEULE with the addition of MBP-WDL4. (**D**) The amount of GST-SYP121ΔC binding to His-KEULE as shown in (C). The values represent mean ± SD from three independent experiments (one-way ANOVA followed by Tukey’s multiple comparisons test, *P* < 0.05). (**E**) LCI assays in *N. benthamiana* leaves. The Luc signals generated by the interaction between cLUC-SYP121 and KEULE-nLUC were detected in the presence of mCherry-WDL4, mCherry-WDL5, or mCherry. The plasmids were co-expressed in *N. benthamiana* leaves at the indicated ratios. Scale bar, 1 cm. (**F**) Quantitative analysis of the Luc intensity as shown in (E). The values were generated from indiGo software and represent mean ± SD for three independent experiments. Significant differences are indicated by letters (one-way ANOVA followed by Tukey’s multiple comparisons test, *P* < 0.05).

It is established that distinct structural domains of SYP121 coordinate its cellular functions (*40*, *47*). To dissect the molecular mechanism underlying the functional connection between SYP121 and WDL4, we proceeded to identify the specific protein domain of SYP121 mediating its interaction with WDL4. SYP121 consists of four domains including an unstructured N-terminal domain (residues 1–39), the Habc domain (residues 40–192), the H3 domain (residues 193–283), and a C-terminal membrane anchor (residues 284–346) (*40*). Using yeast two-hybrid assay, we found that WDL4 interacted with the N terminus of SYP121, but not the HQ fragment of SYP121 containing the Habc domain and Qa-SNARE domain (Fig. 4A). Next, we performed the Co-IP assay using *N. benthamiana* leaves transiently expressing *WDL4-GFP* together with full length *SYP121* or *SYP121*Δ*N*. Immunoprecipitation of WDL4-GFP resulted in the co-purification of full length SYP121, but not SYP121ΔN (Fig. 4B). These results showed that the N terminus of SYP121 is essential for binding of WDL4.

The N terminus of SYP121 is important for regulating the formation of SNARE complex (*47*). In addition, Arabidopsis Sec1/Munc18 (SM) protein KEULE has been reported to associate with the N terminus of SYP121 and regulate SYP121-dependent SNARE assembly through multiple binding modes (*22*, *47*, *48*). To investigate whether WDL4 could affect the interaction between SYP121 and KEULE, we performed the in vitro pull-down assay with gradient concentrations of MBP-WDL4. Increased amount of GST-SYP121ΔC was pulled down by His-KEULE with the addition of MBP-WDL4 (Fig. 4, C and D). Next, we performed the LCI assays in *N. benthamiana* leaves. Luc activity was detected upon co-expression of *KEULE-nLUC* and *cLUC-SYP121* in equal amount. Luc activity resulting from the interaction between KEULE and SYP121 was gradually enhanced when *mCherry-WDL4* was added in equal or double amount. However, this effect was not observed when empty *mCherry* or *mCherry-WDL5* was introduced (Fig. 4, E and F). These results showed that WDL4 enhances the interaction between SYP121 and KEULE, indicating that WDL4 could possibly affect the regulatory interactions of SYP121.

### WDL4 promotes the interaction between SYP121 and VAMP721

**Fig. 5.**
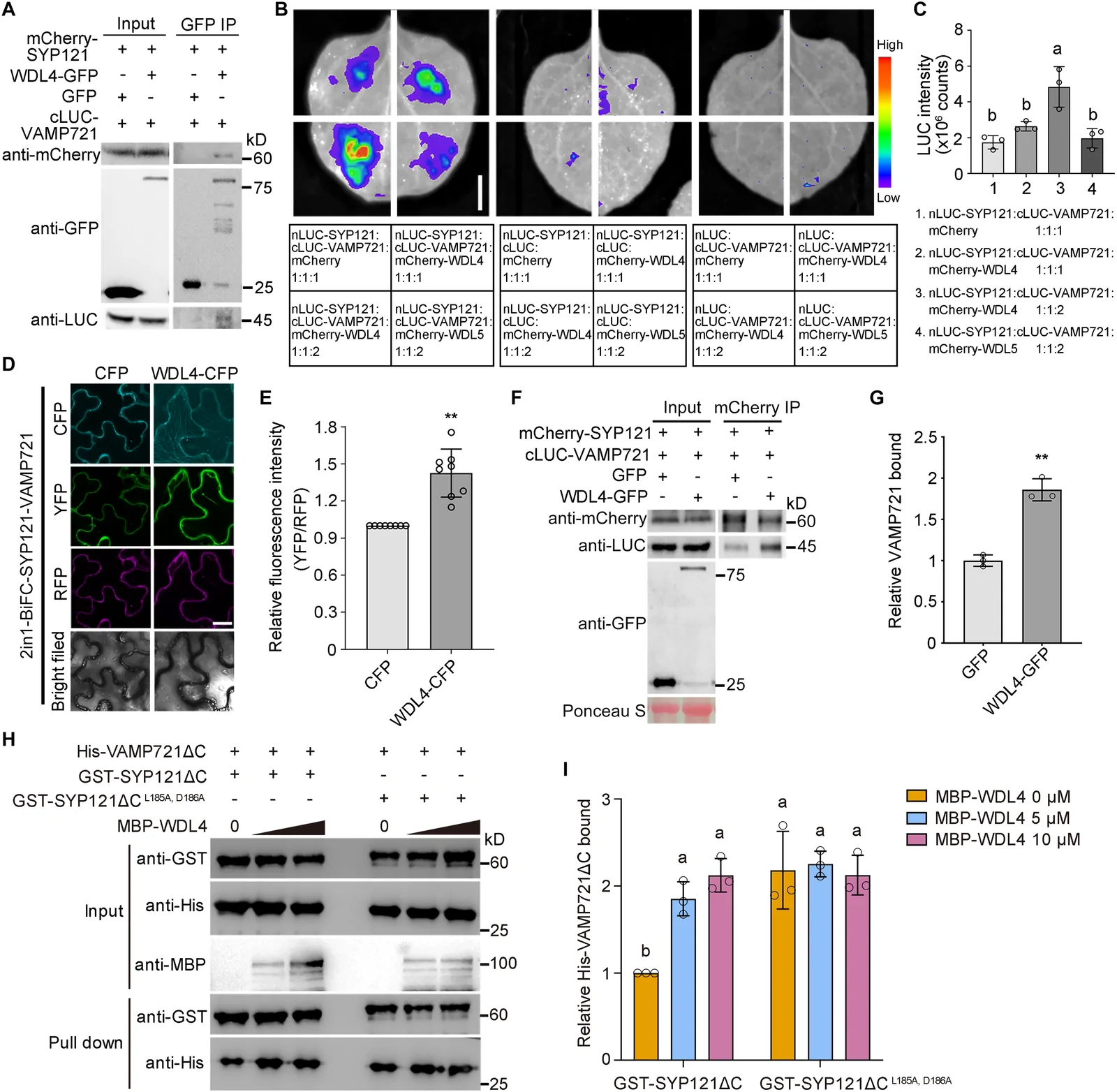
WDL4 promotes the interaction between SYP121 and VAMP721. (**A**) Co-IP assay in *N. benthamiana* leaves. WDL4-GFP and GFP were immunoprecipitated and coimmunoprecipitated proteins were detected with anti-mCherry and anti-LUC antibodies. GFP was used as a negative control. (**B**) LCI assays showing the effect of WDL4 on the SYP121-VAMP721 interaction. cLUC or nLUC was used as negative controls. Scale bar, 1 cm. (**C**) Quantification of the Luc intensity in (B). Values were generated from indiGo software and represent mean ± SD for three independent experiments. Different letters indicate significant differences (one-way ANOVA followed by Tukey’s multiple comparisons test, *P* < 0.05). (**D**) BiFC assays showing the effect of WDL4 on the SYP121-VAMP721 interaction. pBiFCt-2in1 was used to co-express *SYP121* and *VAMP721* for BiFC analysis and to provide RFP fluorescence as the internal control. *WDL4-CFP* or *CFP* were co-expressed. Scale bar, 10 μm. (**E**) Quantification of YFP/RFP fluorescence ratio in (D). Values represent mean ± SD for 8 independent leaves (Student’s *t* test, **P < 0.01). (**F**) Co-IP assay showing the effect of WDL4 on the SYP121-VAMP721 interaction. *mCherry-SYP121* and *cLUC-VAMP721* were co-expressed with *WDL4-GFP* or *GFP*. mCherry-SYP121 was immunoprecipitated and coimmunoprecipitated protein was detected with anti-LUC antibody. (**G**) Quantification of VAMP721 binding to SYP121 in (F) . Values represent means ± SD of three independent experiments with similar results (Student’s *t* test, **P < 0.01). (**H**) In vitro pull-down assay. Affinity-purified GST-SYP121ΔC or GST-SYP121ΔC^L185A, D186A^ was used to pull down His-VAMP721ΔC in the presence of increasing concentration of MBP-WDL4. (**I**) Quantification of His-VAMP721ΔC binding to GST-SYP121ΔC or GST-SYP121ΔC^L185A, D186A^. The values represent mean ± SD of three independent experiments with similar results. Different letters indicate significant differences (one-way ANOVA followed by Tukey’s multiple comparison test, *P* < 0.05).

SYP121 assembles with its cognate R-SNARE VAMP721 to mediate membrane fusion (*21*). Thus, we further examined whether WDL4 could affect the interaction between SYP121 and VAMP721. We found that WDL4 did not interact with VAMP721 in the yeast (fig. S9). However, both SYP121 and VAMP721 were coimmunoprecipitated with WDL4-GFP, but not free GFP through the Co-IP assay (Fig. 5A), indicating that WDL4 is associated with the SYP121-VAMP721 complex. Because WDL4 is a MAP and associates with cortical microtubules through its C-terminal region, we tested whether microtubule integrity affects the WDL4-associated SYP121-VAMP721 complex using the microtubule-depolymerization drug oryzalin. We first confirmed that oryzalin treatment effectively disrupted WDL4-GFP-labeled cortical microtubule structures in *N. benthamiana* leaf epidermal cells (fig. S10A). In Co-IP assays, both SYP121 and VAMP721 were co-immunoprecipitated with WDL4 in DMSO-treated samples. After oryzalin treatment, the relative amounts of SYP121 and VAMP721 co-immunoprecipitated with WDL4 were significantly reduced (fig. S10, B and C), indicating that microtubule depolymerization compromises the formation or stability of the WDL4-SYP121-VAMP721 complex.

To evaluate the effect of WDL4 on the interaction between SYP121 and VAMP721, we first performed the LCI assays in *N. benthamiana* leaves. Luc activity was detected upon co-transformation of *35S: nLUC-SYP121* and *35S: cLUC-VAMP721* in equal amount, consistent with previously established interaction between SYP121 and VAMP721 (Fig. 5B) (*21*). Moreover, Luc activity resulting from the interaction between SYP121 and VAMP721 was gradually enhanced when *mCherry-WDL4* was added in equal or double amount. However, this effect was not observed when *mCherry-WDL5* was introduced (Fig. 5, B and C). The expression levels of *WDL4*, *WDL5*, *nLUC-SYP121* and *cLUC-VAMP721* were determined by RT-qPCR (fig. S11). These results suggest that WDL4 facilitates the interaction between SYP121 and VAMP721. We next verified these results in bimolecular fluorescence complementation (BiFC) assays in *N. benthamiana* leaf epidermal cells using the 2in1 cloning system as reported (*49*). In a single 2in1-BiFC-SYP121-VAMP721 vector, two cassettes expressing *nYFP-SYP121* and *cYFP-VAMP721* were included to ensure their equivalent expression level, and RFP was included as an internal expression control for ratiometric quantification of the interaction intensity (*50*). Consistent with previous report, YFP fluorescence signal was observed when this construct was transiently transformed into *N. benthamiana* leaves, supporting the interaction between SYP121 and VAMP721 (*50*). Upon co-transformation of *WDL4-CFP* but not free *CFP*, YFP fluorescence intensity was evidently enhanced in cells co-expressing nYFP-SYP121 with cYFP-VAMP721 (Fig. 5, D and E). Furthermore, we performed the Co-IP assay to verify the effect of WDL4 on the SYP121-VAMP721 interaction. *WDL4-GFP* (or *GFP*), *mCherry-SYP121* and *cLUC-VAMP721* were co-transformed into *N. benthamiana* leaves, and the extracted proteins were subjected to immunoprecipitation using mCherry-SYP121 as the bait. As shown in Fig. 5, F and G, VAMP721 could be immunoprecipitated by SYP121 as previously reported (*21*), and the level of immunoprecipitated VAMP721 protein was significantly increased when co-expressing WDL4-GFP, as compared to the expression of GFP. Collectively, these results demonstrated that WDL4 promotes the interaction between SYP121 and VAMP721.

Since WDL4 contains separate domains for SYP121 interaction and microtubule association, we further tested whether these domains are sufficient to enhance the SYP121-VAMP721 interaction. In LCI assays, full-length WDL4 enhanced the interaction between SYP121 and VAMP721, whereas WDL4N or WDL4C showed significantly weaker enhancement (fig. S12). The expression levels of the indicated constructs were comparable among the tested groups. Together with the complementation results shown in Fig. 2I, these data indicate that efficient enhancement of SYP121-VAMP721 interaction requires the coordination of both domains in WDL4.

Since the microtubule-associated domain of WDL4 was required for the enhancement of the SYP121-VAMP721 interaction, we further examined whether microtubule integrity is important for this enhancement. In Co-IP assays using mCherry-SYP121 as the bait, the amount of VAMP721 co-immunoprecipitated with SYP121 increased when co-expressing *WDL4-GFP* but not *GFP* alone. This enhancement was strongly reduced by oryzalin treatment (fig. S13). These results indicate that microtubule integrity is important for WDL4 to efficiently promote the SYP121-VAMP721 interaction.

To gain further insight into whether this enhancement depends on the conformational state of SYP121, we used the SYP121ΔC^L185A, D186A^ mutant, in which substitutions in the linker between the Habc and H3 domains favor an open Qa-SNARE conformation (*47*). In vitro pull-down assays showed that MBP-WDL4 increased the amount of His-VAMP721ΔC pulled down by GST-SYP121ΔC, consistent with the results above. The basal interaction between GST-SYP121ΔC^L185A, D186A^ and His-VAMP721ΔC was stronger than that between GST-SYP121ΔC and His-VAMP721ΔC, consistent with the open-like nature of this mutant. Importantly, MBP-WDL4 did not further enhance VAMP721ΔC binding to GST-SYP121ΔC^L185A, D186A^, although MBP-WDL4 still directly interacted with GST-SYP121ΔC^L185A, D186A^ (Fig. 5, H and I, fig. S14). These results suggest that WDL4 enhances SYP121-VAMP721 interaction in a manner that depends on the conformational state of SYP121, consistent with a model in which WDL4 promotes or stabilizes an open-like state of SYP121 for VAMP721 recruitment.

### WDL4 promotes PM accumulation of VAMP721

**Fig. 6.**
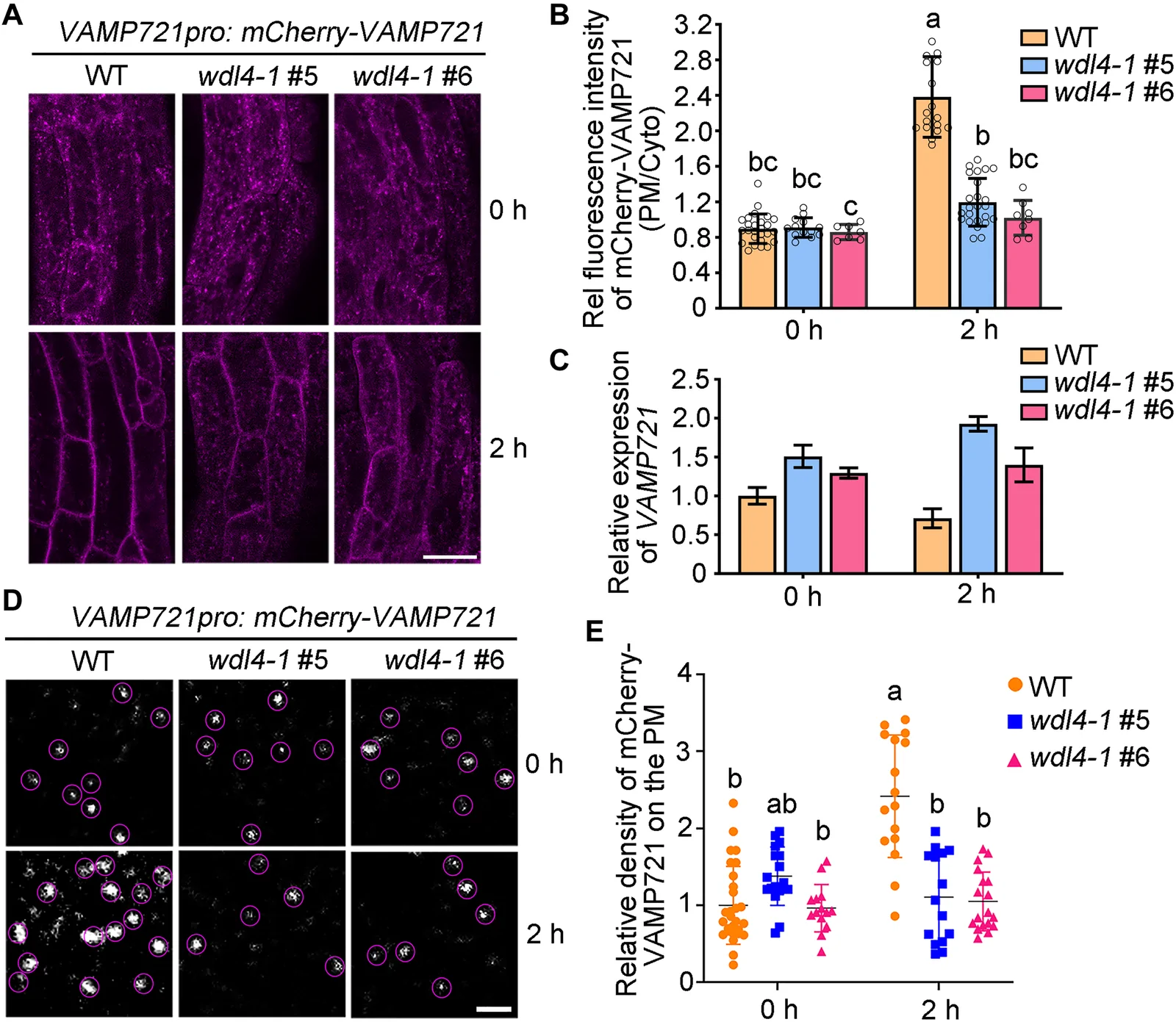
WDL4 promotes VAMP721 translocation to the PM during apical hook opening. (**A**) Representative images of VAMP721 localization in hook epidermal cells in WT and *wdl4–1* seedlings expressing *VAMP721pro: mCherry-VAMP721*. Seedlings were germinated and grown in the dark for 42 h (0 h), followed by white light illumination for 2 h. Light-triggered VAMP721 translocation to the PM was observed in WT, while this effect was obviously attenuate in *wdl4–1* mutants. Scale bar, 20 μm. (**B**) Quantification of light-triggered VAMP721 translocation to the PM as shown in (A). The ratio of fluorescence intensity between the PM-localized VAMP721 and cytosolic VAMP721 (PM/Cyto) was calculated. Values represent mean ± SD (n ≥ 7 seedlings). Significant differences are determined by one-way ANOVA followed by Tukey’s multiple comparisons test. Different letters indicate significant differences at *P* < 0.05. Experiments were repeated three times independently with similar results. (**C**) Relative expression of *VAMP721* in the apical hook of the seedlings used in (A). One to two millimeters of the apical hook regions from seedlings were used for RNA extraction. Values represent mean ± SD for three individual experiment repeats. (**D**) VA-TIRF microscopy imaging showed mCherry-VAMP721 puncta (purple circles) on the PM of hook epidermal cell in WT and *wdl4–1* mutant seedlings. Seedlings were germinated and grown in the dark for 42 h (0 h), followed by white light illumination for 2 h. Scale bar, 1 μm. (**E**) Quantitative analysis of the amount of PM-localized mCherry-VAMP721 puncta in WT and *wdl4–1* seedlings as shown in (D). Values represent mean ± SD (n ≥ 14 cells). Significant differences are determined by Kruskal-Wallis test followed by Dunn’s multiple comparisons test with Bonferroni-adjusted *P* values. Different letters indicate significant differences at *P* < 0.05. Experiments were repeated three times independently with similar results.

VAMP721-positive vesicles have been implicated in PIN protein trafficking from the TGN to the PM, as previously reported (*25*). Considering the roles of WDL4 in both regulating PIN protein trafficking and enhancing SYP121-VAMP721 interaction (Fig. 5) (*44*), we hypothesized that WDL4 might promote the PM accumulation of VAMP721 during apical hook opening. To test this hypothesis, we crossed a transgenic line expressing *VAMP721pro: mCherry-VAMP721* (*51*) with the *wdl4–1* mutant, and observed the cellular localization of mCherry-VAMP721 in the epidermal cells of hook region upon light treatment. mCherry-VAMP721 predominantly localized to punctate structures in the cytoplasm in both WT and *wdl4–1* mutant when seedlings were grown in the dark for 42 h (Fig. 6A) (*51*). Interestingly, the PM localization of mCherry-VAMP721 was significantly enhanced in WT after light illumination for 2 h. However, this effect was obviously attenuated in the *wdl4–1* mutant, with more punctate mCherry-VAMP721 remaining in the cytoplasm (Fig. 6, A and B). Subsequent RT-qPCR analysis showed that the observed differences in the localization of mCherry-VAMP721 were not caused by its transcription level (Fig. 6C). Moreover, PM-associated mCherry-VAMP721 signals were observed by VA-TIRFM. The density of mCherry-VAMP721 foci on the PM was significantly increased in WT after light illumination for 2 h, while it remained similar in *wdl4–1* mutant (Fig. 6, D and E).

We next tested whether microtubule integrity is important for this light-induced PM accumulation of VAMP721. Oryzalin treatment strongly reduced the light-induced PM accumulation of mCherry-VAMP721 in WT seedlings, but had little additional effect in the *wdl4–1* mutant (fig. S15). Consistently, VA-TIRFM showed that oryzalin treatment reduced the density of VAMP721-associated foci at the PM (fig. S15). Together, these results indicate that WDL4 is required for efficient light-induced VAMP721 PM accumulation, and that microtubule integrity is important for this process.

## DISCUSSION

**Fig. 7.**
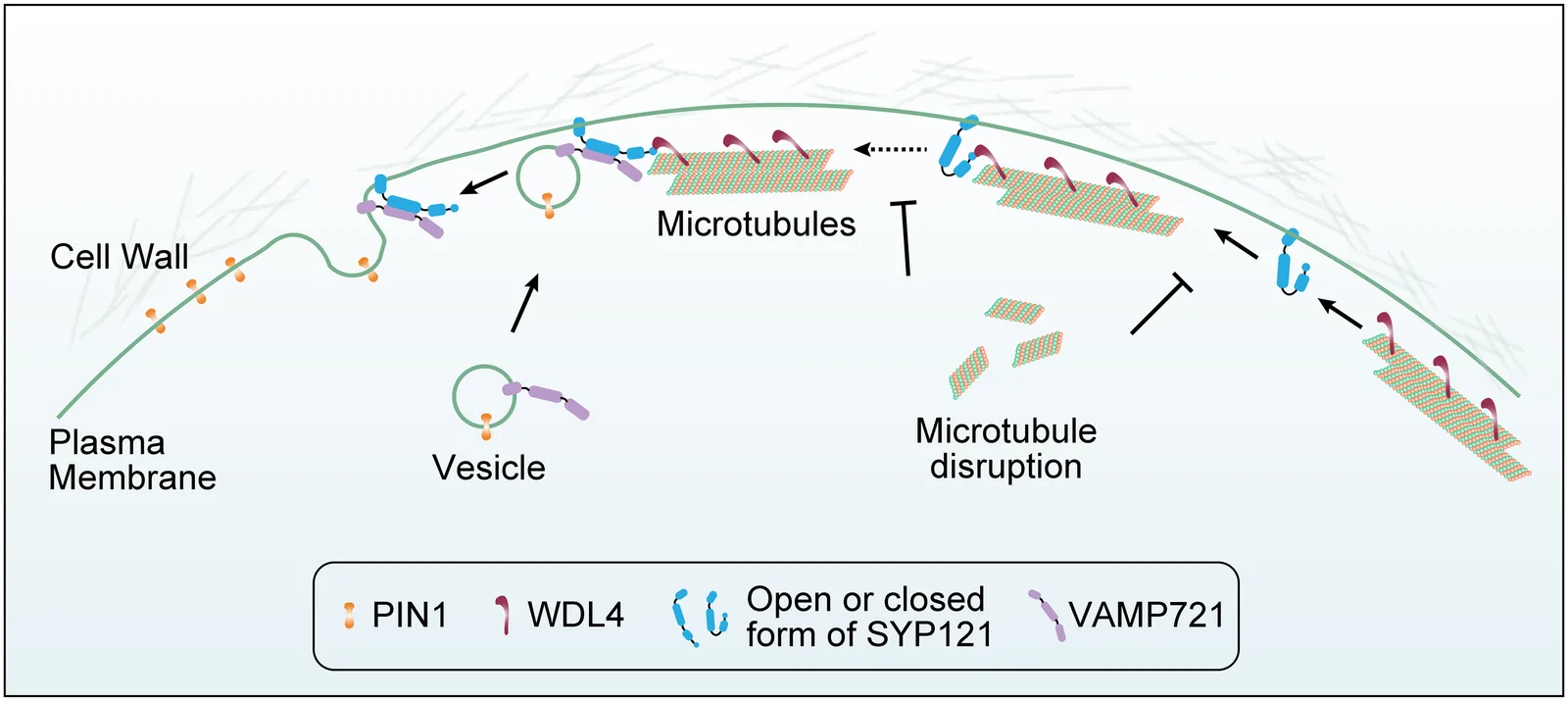
A proposed working model of the WDL4-SYP121 module in regulating auxin-regulated growth remodeling. Experimental evidence shows that the N-terminal region of WDL4 mediates its interaction with SYP121 and the C-terminal region of WDL4 associates with cortical microtubules. WDL4 interacts with the N terminus of SYP121, and enhances the interaction between SYP121 and its cognate R-SNARE VAMP721. WDL4 is also required for efficient light-induced accumulation of VAMP721 at the PM. Microtubule disruption compromises WDL4-associated SNARE complex formation, WDL4-mediated enhancement of the SYP121-VAMP721 interaction, and VAMP721 PM accumulation. Based on analyses using an open-like SYP121 mutant, we propose that WDL4 may promote or stabilize a SYP121 state favorable for VAMP721 recruitment. Together, these findings support a working model in which WDL4 coordinates cortical microtubule with SYP121-associated trafficking, thereby contributing to PIN1 recycling back to the PM and auxin transport-dependent apical hook opening.

Auxin-dependent growth remodeling requires dynamic PM distribution of auxin transporters through vesicular trafficking. Although cortical microtubules are positioned immediately beneath the PM and have been implicated in trafficking events, how they interface with PM-associated SNARE machinery remains unclear. Here, we identify WDL4 as a microtubule-associated regulator that links cortical microtubules with the SYP121-VAMP721 SNARE complex during apical hook opening. We demonstrate that WDL4 physically and genetically interacts with SYP121, and links SYP121 to cortical microtubules through separate SNARE-interaction and microtubule-binding domains. Moreover, WDL4 enhances the interaction between SYP121 and its cognate R-SNARE VAMP721, and promotes the efficient PM accumulation of VAMP721. Disruption of either WDL4 or SYP121 impaired PM accumulation and recycling of the auxin efflux carrier PIN1, and attenuated auxin-dependent apical hook opening. Collectively, these results support a working model in which WDL4-SYP121 interaction links cortical microtubule to SNARE-mediated PM trafficking, thereby contributing to auxin transport-dependent hook opening (Fig. 7).

A key question is the molecular mechanism by which WDL4 regulates SYP121-VAMP721 pairing and the associated trafficking process. Our results suggest that WDL4 does not simply act as a passive scaffold between SYP121 and VAMP721. WDL4 binds the N terminus of SYP121, which has been implicated in the regulation of SNARE complex assembly (*22*, *47*). Consistently, WDL4 enhanced not only the SYP121-VAMP721 interaction but also the interaction between SYP121 and the SM protein KEULE/SEC11, suggesting that WDL4 may affect regulatory interactions of SYP121. Previous studies have shown that KEULE could interact with SYP121 in two distinct modes, one requiring only KEULE and SYP121, and the second depending on the assembled SYP121-VAMP721-SNAP33 SNARE complex (*47*). Moreover, the open-like SYP121ΔC^L185A, D186A^ mutant showed increased binding with GST-KEULE in pull-down assays, suggesting that KEULE binding is influenced by the conformational state of SYP121 (*47*). In our study, WDL4 enhanced VAMP721ΔC binding to SYP121ΔC but did not further enhance VAMP721ΔC binding to the open-like SYP121ΔC^L185A, D186A^ mutant, although WDL4 could still interact with this mutant protein. These results are more consistent with a model in which WDL4 promotes or stabilizes an open-like state of SYP121, thereby facilitating cognate SNARE recruitment and potentially favoring KEULE engagement. Post-translational modifications are important regulatory mechanisms for membrane trafficking. Recent work showed that LKS4-mediated phosphorylation of SYP121 at T270 promotes its interaction with VAMP722 and SNARE complex assembly (*52*). In our in vitro pull-down assays, purified recombinant WDL4 enhanced the SYP121-VAMP721 interaction in the absence of plant-specific post-translational modification machinery. However, we do not exclude the possibility that post-translational modifications may contribute to WDL4-associated SNARE assembly regulation in vivo.

Beyond promoting efficient light-induced PM accumulation of VAMP721, WDL4 may also affect the trafficking behavior of SYP121 itself. PEN1/SYP121 has been shown to undergo dynamic trafficking between the PM and intracellular compartments in a BFA-sensitive manner (*53*, *54*). Moreover, active ROP2 promotes both SYP121-VAMP722 SNARE complex formation and the exocytic recovery of SYP121 at the apical PM during root hair tip growth (*24*). These studies suggest that the trafficking of SYP121 itself is dynamically regulated. Consistently, our BFA washout assays showed that the number of GFP-SYP121-labeled BFA bodies was less efficiently reduced in *wdl4–1* than in WT seedlings after BFA removal (fig. S16), suggesting that WDL4 contributes to the dynamic trafficking of SYP121.

Our results show that intact cortical microtubules are important for the function of WDL4 in regulating SNARE pairing and VAMP721 PM accumulation. WDL4 contains separate domains for SYP121 interaction and cortical microtubule association, but neither WDL4N nor WDL4C alone was sufficient to fully enhance the SYP121-VAMP721 interaction or rescue the hook-opening defect in the *wdl4* mutant. Moreover, microtubule depolymerization affected the formation or stabilization of WDL4-SYP121-VAMP721 complex and WDL4-mediated enhancement of the SYP121-VAMP721 interaction, and attenuated light-induced VAMP721 PM accumulation. Together, these results support a model that WDL4 acts as a microtubule-associated regulator that coordinates microtubule-dependent spatial organization with the regulation of SYP121-VAMP721 pairing.

Our data further connect the WDL4-SYP121 module with auxin transport-dependent differential growth. BFA treatment and washout assays showed that the reduced PM accumulation of PIN1 in the *wdl4* and *syp121* mutants is mainly associated with defective PIN1 recycling rather than internalization. Together with the altered DR5 reporter patterns, reduced concave-side epidermal cell length, and enhanced NPA sensitivity, these results support a functional link between WDL4-SYP121-associated trafficking and auxin transport-dependent hook opening. IAA treatment did not detectably affect WDL4-GFP localization in the apical hook region, WDL4 transcript or protein levels, or the WDL4-SYP121 interaction (fig. S17). These results indicate that the WDL4-SYP121 module may function mainly as part of the trafficking machinery that supports auxin transporter distribution during apical hook opening, rather than as an auxin-induced component. Although PIN1 was used as a representative trafficking output in this study, we do not intend to suggest that the WDL4-SYP121 module is specific only to PIN1. SYP121 and VAMP721/722 participate in the trafficking of multiple PM cargoes, and auxin-dependent developmental processes involve multiple PIN proteins and AUX1/LAX influx carriers. Therefore, broader effects on other auxin transporters or additional SYP121-dependent cargoes remain possible.

Previous work has established a role for microtubules in endosomal sorting and recycling during auxin transporter trafficking, demonstrated by CLASP-mediated association between microtubules and SNX1-endosomes (*36*). We did not detect direct interaction between CLASP and SYP121 or between WDL4 and SNX1 in yeast two-hybrid assays (fig. S18), suggesting that WDL4-SYP121 and CLASP-SNX1 may represent distinct microtubule-associated trafficking modules. The CLASP-SNX1 module is linked to endosomal sorting, whereas the WDL4-SYP121 module described here is linked to SNARE-mediated PM delivery during cargo recycling. However, indirect or context-dependent cross-talk between these pathways cannot be excluded. Together, these findings suggest that microtubule-associated regulation can operate at multiple stages of the trafficking process. In addition, other SNARE proteins have also been reported to be involved in PIN1 trafficking and recycling, including VAMP714 and SYP132 (*19*, *55*). We did not detect direct interaction between WDL4 and VAMP714 or SYP132 in yeast two-hybrid assays (fig. S19), suggesting that WDL4 does not broadly interact with all SNARE proteins linked to PIN1 trafficking. Nevertheless, we do not exclude possible interactions with other SNAREs not tested here.

To determine whether the SYP121-WDL4 module may also function in other auxin-dependent growth processes, we examined lateral root (LR) development (*56*). As shown in fig. S20, loss-of-function mutants of *SYP121* and *WDL4* showed reduced LR density, which was rescued by the corresponding complementation lines. The *syp121–5*, *wdl4–1*, and *syp121–5 wdl4–1* mutants also showed a lower ratio of emerged lateral roots. Lateral root emergence largely relies on PIN-mediated auxin transport to promote auxin accumulation in the developing primordia and the overlying cell layers (*56*, *57*). Consistent with the emergence defect, DR5: GFP signals were reduced in LR primordia and overlying cell layers, and the fluorescence intensity of PIN1-GFP at the PM was decreased in *syp121–5*, *wdl4–1*, and *syp121–5 wdl4–1* mutants (fig. S20). These supplementary results suggest that the SYP121-WDL4 module may also contribute to the PM accumulation of PIN1 during LR emergence. Together, our study identifies a microtubule-associated MAP-SNARE module that links cortical microtubule with SYP121-VAMP721-associated trafficking, thereby supporting auxin transporter redistribution and auxin-dependent growth remodeling.

## MATERIALS AND METHODS

### Plant growth conditions and materials

The sterilized seeds were sowed on 0.8% agar plates containing half-strength Murashige and Skoog (MS) salts and 1% sucrose (pH 5.8), then vernalized in the dark at 4°C for 2 days. Following this step, the plates were placed in white light (116 μmol m^−2^ s^−1^) at 22°C for 18 h to promote germination. Next, the seedlings were grown vertically in the dark for the indicated time points at 22°C.

The Arabidopsis (*A. thaliana*) accessions Ws and Col-0 were used as WT in this study. The *DR5: GFP* (*46*) and *PIN1pro: PIN1-GFP* (*46*) transgenic lines were kindly provided by Prof. Jing Zhang of China Agricultural University. The *VAMP721pro: mCherry-VAMP721* transgenic line (*51*) was kindly provided by Prof. Jinxing Lin of Beijing Forestry University. The T-DNA insertion knockdown mutant *syp121–5* (SALK_087016) (*24*), *35S: GFP-SYP121* transgenic line (*58*) and *SYP121pro:mCherry-SYP121* were kindly provided by Prof. Ying Fu of China Agricultural University. The *wdl4–1* mutant, *wdl4–2* mutant, the complementary lines, *Super: mCherry-WDL4*, *wdl4–1 DR5: GFP* and *wdl4–1 PIN1pro: PIN1-GFP* transgenic lines were used as previously reported (*44*).

The *syp121–7* mutant line was obtained by CRISPR/Cas9 technology as described previously (*38*). The target site was selected from the first exon of At3g11820. Following genome sequencing analysis, line 50 harbored a nucleotide insertion into the first exon of *SYP121* resulting in premature termination of the protein translation, which was used for further phenotypic characterization.

To complement the hook phenotype of the *syp121–5* and *syp121–7* mutants, *SYP121* native promoter (824-bp upstream of the initiation codon ATG) and its CDS in frame with *GFP* were ligated into the pCAMBIA1390 vector, then transformed into the *syp121–5* and *syp121–7* mutants by floral dipping with Agrobacterium (*Agrobacterium tumefaciens*) strain GV3101 pMP90. Homozygous T_3_ transgenic lines were used for further analyses.

The *syp121–5 wdl4–1* double mutant was generated by genetically crossing *wdl4–1* with *syp121–5* and the F_2_ population was characterized by PCR-based genotyping. Moreover, genetic crosses generated *Super: mCherry-WDL4* × *35S: GFP-SYP121*, *WDL4pro: WDL4-GFP* × *SYP121pro: mCherry-SYP121*, *wdl4–1 VAMP721pro: mCherry-VAMP721*, *wdl4–1 35Spro: GFP-SYP121*, *syp121–5 DR5: GFP*, *syp121–5 wdl4–1 DR5: GFP*, *syp121–5 PIN1pro: PIN1-GFP* and *syp121–5 wdl4–1 PIN1pro: PIN1-GFP* lines with the genotypes verified using PCR- or mCherry (or GFP)-based assays.

To generate transgenic lines *wdl4–1 WDL4pro: WDL4N-GFP* and *wdl4–1 WDL4pro: WDL4C-GFP*, the native *WDL4* promoter (1,915-bp upstream of the initiation codon ATG) and *WDL4N* (or *WDL4C*) CDS in frame with *GFP* were PCR-amplified and inserted into the pCAMBIA1390 vector. Then these constructs were transformed into the *wdl4–1* mutant, respectively. Two independent transgenic lines of homozygous T_3_ were used for further study.

### Phenotypic analysis

Representative images of hook curvature were acquired using an Olympus SEX16 (10× objective, 2× magnification), and the hook angles were measured by Fiji (ImageJ) v.2.0. software, as described previously (*9*, *43*). For light-induced rapid hook opening system, etiolated seedlings were grown vertically for 42 h after germination, then exposed to white light (116 μmol m^−2^ s^−1^) at 22°C for 1–3 h, as described previously (*44*). The apical hook phenotype was recorded at the designated time points and measured using Fiji (ImageJ) v.2.0. software. For NPA (Sigma-Aldrich; 10 mM stock in DMSO) treatments, the seedlings were exposed to white light for 4 h and recorded for apical hook measurement.

For lateral root (LR) phenotype analysis, seedlings were grown on half-strength MS medium supplemented with 1% (w/v) sucrose and 0.8% (w/v) agar under continuous white light at 22°C for 5 days, and then transferred to fresh medium for an additional 5 days. Primary root lengths were measured using Fiji (ImageJ) v.2.0. software, and the number of LR was manually counted. Lateral root density (LR density) was defined as the number of LR per unit length of primary root (LRs/cm).

For observation of non-emerged lateral root primordia (nLRPs) and emerged lateral roots (LREs), seedlings were treated according to a standardized ethanol dehydration protocol prior to microscopic examination (*59*). Subsequently, seedlings were mounted in 50% glycerol solution and imaged using an OLYMPUS BX51 differential interference contrast (DIC) microscope equipped with a 20× objective lens (*60*). Quantification was performed through manual classification and counting.

### RNA extraction and RT-PCR analysis

Total RNA was extracted from dark-grown whole seedlings or 1 to 2 mm of apical hypocotyl (excluding cotyledons) with an RNA purification kit (Bio Teke, Beijing, China). First-strand cDNAs were synthesized from total RNA (1 μg) using oligo dT primers and M-MLV reverse transcriptase (TaKaRa, Shiga, Japan). RT-qPCR was performed to determine *SYP121*, *PIN1*, *VAMP721*, *nLUC*, *cLUC*, *GFP*, *IAA1*, *WDL4* and *WDL5* relative transcript levels using an ABI 7500 RT PCR detection system with SYBR Premix ExTaq Mix (TaKaRa), according to the manufacturer’s instructions. For detection expression of *SYP121*, *PIN1*, *VAMP721*, *IAA1* and *WDL4* in Arabidopsis seedlings, the *EF1a* gene was used as an internal control. In the LCI assay to detect the expression levels of *nLUC*, *cLUC*, *GFP*, *WDL4* and *WDL5*, total RNA was extracted from *N. benthamiana* leaves and the *18S rRNA* gene was used as an internal control. The RT-qPCR primers are listed in table S1.

### Ballistics-mediated transient expression in leaf epidermal cells

To visualize the cellular localization of WDL4N-GFP and WDL4C-GFP, ballistics-mediated transient expression was performed in Col-0 leaf epidermal cells as previously published methods (*61*). We used 2.5 μg of plasmid (*35S:WDL4-GFP /35S:WDL4N-GFP*/*35S:WDL4C-GFP*) for particle bombardment. Then, the GFP signal was detected in leaf epidermal cells after the seedlings grown in the dark at 22°C for 6–8 h using confocal microscopy (Leica Stellaris 5, 40× oil objective). The primers used for plasmid construction are listed in table S1.

### In vitro microtubule binding assay

Porcine brain tubulin was purified and labeled with Rhodamine as previously described (*62**,* *63*) and microtubules were prepared according to previously published protocols (*64*). The purified proteins were centrifuged at 100,000g for 15 min at 4°C before use. Rhodamine-labeled and paclitaxel-stabilized microtubules were incubated with various fusion proteins in PEMT buffer (10 μM paclitaxel, 1 mM MgCl_2_, 1 mM EGTA, and 100 mM PIPES-KOH, pH 6.9) at room temperature for 10 min. The reactions were then fixed with 1% (v/v) glutaraldehyde for observation and imaging using confocal microscope (Leica SP8, 100× oil objective).

### Yeast two-hybrid assay

The coding sequences of the cytosolic fragment of *SYP121* (*SYP121*Δ*C*), *SYP121N* (residues 1–39), and *SYP121HQ* (residues 40–283) were cloned into vector pGBKT7 (containing the GAL4 DNA binding domain sequence) (*40*). The CDS of *WDL4*, *WDL4N* (residues 1–218), *WDL4C* (residues 219–432) and *WDL5* were inserted into pGADT7 vector (containing the GAL4 activation domain sequence). Yeast transformation was performed according to the manufacturer’s protocol (Clontech, Mountain View, CA, USA). The paired vectors were co-transformed into the yeast strain AH109 cells, and selected on synthetic dropout medium (SD/−Leu-Trp). Individual colonies were then picked and spotted onto SD/−Leu-Trp and SD/−Leu-Trp-His containing 3 mM or 5 mM 3-AT (3-amino-1,2,4-triazole) as indicated in the corresponding legends. Plates were incubated at 28°C for 2–4 days before imaging.

To test for the interaction between WDL4 and VAMP721, the coding sequences of the cytosolic fragment of *VAMP721* (*VAMP721*Δ*C*) was cloned into vector pGBKT7 (*65*). The CDS of *SYP121ΔC* was inserted into pGADT7 vector. The VAMP721ΔC-BD and SYP121ΔC-AD were co-transformed as a positive control.

To test for the interaction between WDL4 and SYP132 or VAMP714, the coding sequences of the cytosolic fragment of *SYP132* (*SYP132ΔC*, residues 1–270) (*66*) or *VAMP714* (*VAMP714ΔC*, residues 1–190) (*19*) was cloned into vector pGBKT7. These plasmids were co-transformed with WDL4-AD. The pair of SYP121ΔC-BD and WDL4-AD was used as a positive control. The primers used for plasmid construction are listed in table S1.

### In vitro pull-down assay

*MBP-WDL4*, *GST-SYP121*Δ*C*, *GST-SYP121ΔC^L185A, D186A^* (*47*), *His-VAMP721*Δ*C* and *His-KEULE* plasmids were generated and transformed into *E. coli* BL21. The expression was induced with 0.8 mM isopropyl-β-D-thiogalactopyranoside (IPTG) at 25°C for 4 h. Following cell sonication, proteins were purified by amylose resin beads (BioLabs, E8022S), Glutathione Sepharose 4B agarose (FORSCIENCE, F042), and Ni-NTA agarose (FORSCIENCE, F041) respectively. All of these proteins were dialyzed with buffer containing 150 mM NaCl, 2.5% glycerol, and 100 mM Tris-HCl, pH 8.0.

For the WDL4-SYP121 interaction assay, 5 μM of GST, GST-SYP121ΔC or GST-SYP121ΔC^L185A, D186A^ was pre-incubated with Glutathione-Sepharose 4B agarose in binding buffer containing 100 mM Tris, pH 8.0 for 2 h at 4°C. Then, equimolar amounts of MBP-WDL4 were added to the beads. The mixtures were incubated with gentle rotation for 2 h at 4°C. Thereafter, collected resin was washed three times with washing buffer containing 100 mM Tris-HCl, pH 8.0, 150 mM NaCl, 1 mM DTT, and 0.05% Tween 20 at 4°C. Bound proteins were eluted and analyzed by immunoblotting using anti-GST (ABclonal, AE006, 1:5000) and anti-MBP (New England Biolabs, E8031S, 1:5000 dilution) antibodies. GST was used as a negative control.

To test the effect of WDL4 on the interaction between SYP121 and KEULE, purified His-KEULE was immobilized on Ni-NTA agarose beads and incubated with GST-SYP121ΔC in the presence of increasing concentrations of MBP-WDL4. The final concentrations of His-KEULE and GST-SYP121ΔC in the reaction were both 4 μM. After reaction for 2 h at 4°C, each resin was washed three times with washing buffer described above at 4°C. Beads were resuspended in 1 × SDS loading buffer, then boiled for 5 min. Samples were analyzed by immunoblotting using a 1:5000 dilution of anti-MBP antibody, a 1:5000 dilution of anti-GST antibody and a 1:5000 dilution of anti-His antibody (ABclonal, AE003), respectively.

To test the effect of WDL4 on the interaction between SYP121 and VAMP721, purified GST-SYP121ΔC or GST-SYP121ΔC^L185A, D186A^ was immobilized on Glutathione Sepharose 4B agarose beads and incubated with His-VAMP721ΔC in the presence of increasing concentrations of MBP-WDL4. The final concentrations of His-VAMP721ΔC and GST-SYP121ΔC in the reaction were both 5 μM. The pull-down assays and immunoblotting were performed similarly. Band intensities were quantified using Fiji (ImageJ) v.2.0. software. The primer sequences for plasmid construction are listed in table S1.

### Luciferase complementation imaging (LCI) assay

For the WDL4-SYP121 interaction assay, the CDS of *WDL4* or *WDL5* was introduced to 35S: nLUC (*67*) vector to generate *35S: WDL4-nLUC* or *35S: WDL5-nLUC* and the CDS of *SYP121* was cloned to 35S: cLUC (*67*) vector to produce *35S: cLUC-SYP121*. Corresponding construct pairs were transformed into *A. tumefaciens* GV3101 pMP90 and co-infiltrated into *N. benthamiana* leaves. After 2 d, LCI assays were performed as described (*67*). A charge-coupled device (CCD; 1300B, Roper Scientific, Trenton, NJ, USA) camera was used to capture the LUC signal.

For LCI assays testing the effect of WDL4 on the SYP121-VAMP721 interaction, *35S: nLUC-SYP121* and *35S: cLUC-VAMP721* were co-infiltrated with different combinations of effector constructs expressing *mCherry-WDL4* at the indicated ratios, as previously described (*68*). mCherry and mCherry-WDL5 served as negative controls. Plants were grown at 28°C for 2 d. LUC activity was detected using LB985 NightShade with indiGo software (Berthhold Tech).

To examine the effect of WDL4 truncations, the CDS of *WDL4N* (residues 1–218) and *WDL4C* (residues 219–432) were separately cloned into the pCAMBIA1300 vector under the control of the *Super* promoter. *Super: WDL4-GFP*, *Super: WDL4N-GFP* or *Super: WDL4C-GFP* constructs were co-infiltrated with *35S: nLUC-SYP121* and *35S: cLUC-VAMP721*. GFP served as a negative control.

The same procedure was used to determine the effect of WDL4 on interaction between SYP121 and KEULE. The CDS of *KEULE* was introduced to 35S: nLUC vector to generate *35S: KEULE-nLUC*.

The primer pairs used for plasmid construction are shown in table S1.

### Co-IP assay

The full-length CDS of *SYP121* and *WDL4* were separately in frame with *mCherry* or *GFP*, which were cloned into the pCAMBIA1300 vector under the control of the *Super* promoter (Invitrogen).

For investigating interaction between WDL4 and SYP121 in vivo, *mCherry-SYP121* and *WDL4-GFP* were transiently co-transfected into *N. benthamiana* leaves by infiltration. After 48 h cultivation, samples were frozen in liquid nitrogen and equal weights of ground tissue were suspended in 500 μL 2 × protein extraction buffer (100 mM Tris-HCl, 300 mM NaCl, 1 mM EDTA, 10% glycerol, 1% NP-40, and 1 × complete protease inhibitor cocktail, pH 7.5). Extracts were maintained on ice for 10 min and separated via centrifugation at 13,000g for 20 min at 4°C. Supernatants were incubated with GFP mAb-Agarose beads (ABclonal, AE079) for 2 h at 4°C with gentle agitation. Co-IP products were washed five times with PBS buffer (140 mM NaCl, 2.7 mM KCl, 1.8 mM KH_2_PO_4_ and 10 mM Na_2_HPO_4_, pH 7.4) at 4°C. Proteins were eluted from the beads by boiling with 2× SDS loading buffer and then detected via immunoblot analysis. Both anti-GFP (ABclonal, AE012) and anti-mCherry (Agnsera, AS163967) antibodies were used at 1:5000 dilutions. GFP was used as a negative control.

To detect the effect of WDL4 on SYP121-VAMP721 interaction in vivo, *mCherry-SYP121* and *cLUC-VAMP721* were co-expressed in *N. benthamiana* leaves in the presence of *WDL4-GFP* or *GFP*. IP was performed using mCherry mAb-Agarose beads (ABclonal, AE073) for 2 h at 4°C. WDL4 and VAMP721 were detected by immunoblotting using anti-GFP and anti-LUC (SIGMA, L0159, 1:5000) antibodies, respectively. GFP was used as a negative control. To examine the effect of oryzalin or IAA treatment, agroinfiltrated leaves were treated 36 h after infiltration. For each leaf, one half was infiltrated with oryzalin or IAA at the indicated concentration and duration, and the other half was infiltrated with the corresponding mock buffer as a control. For oryzalin treatment, 10 μM oryzalin was infiltrated for 6 h before protein extraction. For IAA treatment, 25 μM IAA was infiltrated for 1 h before protein extraction.

To test the protein domain of WDL4 mediating its interaction with SYP121 in vivo, WDL4N-GFP, WDL4C-GFP, WDL4-GFP and GFP were used in the experiment.

To test the protein domain of SYP121 mediating its interaction with WDL4 in vivo, the CDS of *SYP121* excluding its unstructured N-terminal domain (residues 1–39) was inserted into the 35S: cLUC vector to produce *cLUC-SYP121*Δ*N*. The coimmunoprecipitated cLUC fusion proteins were detected using an anti-LUC antibody. GFP was used as a negative control.

The primer sequences for plasmid construction are listed in table S1.

### BiFC assay

To test the effect of WDL4 on the interaction between SYP121 and VAMP721, we performed bimolecular fluorescence complementation (BiFC) assays in *N. benthamiana* leaf epidermal cells by the 2in1 system, which contains a set of independent Gateway-compatible cassettes with 35S promoters (*49*). In the pBiFCt-2in1-NC vector, two cassettes respectively include *nYFP-SYP121* and *cYFP-VAMP721* constructs and the third cassette includes the coding sequence for red fluorescent protein (RFP) as an internal expression control to ratiometric quantification of BiFC (*50*). The pBiFCt-2in1-NC vector was kindly provided by Prof. Ruixi Li of Southern University of Science and Technology. The CDS of *WDL4* was introduced to UBQ10: CFP vector to generate *UBQ10: WDL4-CFP.* Then construct pairs pBiFCt-2in1-NC + WDL4-CFP and 2in1-NC + CFP were respectively co-infiltrated into *N. benthamiana* leaves. The plants were allowed to recover for 2 days at 28°C. Fluorescent YFP/RFP signals were observed using confocal microscope (Leica Stellaris 5, 40× oil objective). Fluorescent intensities of YFP/RFP signal were measured by Fiji (ImageJ) v.2.0. software.

The primers used for generating the constructs are shown in table S1.

### Scanning Electron Microscopy

Dark-grown 3-day-old seedlings were mounted on a sample holder. The chamber was evacuated after the sample was placed. After auto-focusing, images were captured with 100× magnification using HITACHI (TM4000Plus, Japan). The apical hook epidermal cell length was measured by Fiji (ImageJ) v.2.0. software.

### Live-Cell Imaging

The *DR5: GFP* expression pattern was photographed by confocal microscopy (Carl Zeiss 880, 10× objective). Quantification and measurement of *DR5: GFP* fluorescence intensity at the concave side of the apical hook were performed as previously described (*44*). For lateral root primordia, the LRP region and the overlying endodermal cells were manually outlined as separate regions of interest (ROIs), and the mean GFP fluorescence intensity was measured for each ROI using Fiji (ImageJ) v.2.0. software.

The subcellular distribution and localization of PIN1-GFP in the apical hook region were observed and measured as described previously (*44*). For lateral root primordia, cells with clearly defined cell boundaries and PM-localized PIN1-GFP signals were selected for quantification using Fiji (ImageJ) v.2.0. software.

BFA treatment and washout assays were performed as previously described (*44*). For BFA treatment, seedlings were pre-incubated in half-strength MS liquid medium containing 50 μM CHX (Sigma-Aldrich, 10 mM stock solution in DMSO) for 60 min, and then treated with 100 μM BFA plus 50 μM CHX for 30 min under white light. For the BFA washout assay, seedlings were pretreated with 50 μM CHX in half-strength MS medium for 30 min, followed by incubation with 100 μM BFA + 50 μM CHX for 1.5 h to induce BFA body formation. Seedlings were rinsed three times and transferred to half-strength MS medium supplemented with 50 μM CHX for a 30 min or 1 h recovery period. All procedures were performed under white light. BFA bodies in the apical hook region were imaged using a spinning-disk confocal microscope with a 40× objective, and autofluorescence signals were collected in a separate channel. All images were acquired under identical instrumental parameters. The number and size of BFA bodies per cell were quantified using Fiji (ImageJ) v.2.0. software, and relative values were normalized to the initial time-point of each seedling and presented as percentages.

The localization of mCherry-VAMP721 in the epidermis cells of hook region was visualized in dark grown 3-day-old seedlings by confocal microscopy (Leica Stellaris 5, 40× oil objective, 1.5× magnification). For the quantification of the PM/cytosol fluorescence intensity ratio, images were analyzed using Fiji (ImageJ) v.2.0. software. Epidermal cells in the apical hook region that were fully captured in the image were used for quantification. For each cell, a line was drawn along the PM to measure the mean PM fluorescence intensity (PM). The whole cytoplasmic region of the same cell was selected to measure the mean cytosolic fluorescence intensity (Cyto). The PM/Cyto ratio was calculated for each cell as the mean PM fluorescence intensity divided by the mean cytosolic fluorescence intensity. Ratios from multiple cells and independent seedlings were used for statistical analysis.

For VA-TIRFM analysis of VAMP721 at the PM, hook epidermal cells of dark-grown 3-d-old transgenic plants expressing mCherry-VAMP721 were observed and recorded with an Olympus IX-81 TIRF microscope (Yokogawa, 150× oil-immersion TIRF objective). The 561-nm laser illumination angle was adjusted to optimize detection of signals near cell surface with minimal background. The number of mCherry-VAMP721 foci at the PM were counted from the captured images via Fiji (ImageJ) v.2.0. software using the Analyze-Particles function.

For the co-localization between WDL4 and SYP121 at the PM, the epidermis cells of hook region from dark grown 3-d-old seedlings were used for VA-TIRF imaging. Images of transgenic plants expressing GFP-SYP121 (mCherry-SYP121) and mCherry-WDL4 (WDL4-GFP) were captured with an Olympus IX-81 TIRF microscope (Yokogawa, 150× oil-immersion TIRF objective). GFP- and mCherry-fused proteins were excited with the 488 nm and 561 nm laser line, respectively. Laser illumination angle was adjusted to optimize the detection of SYP121 signals near cell surface with minimal background. Time-lapse images were acquired at fixed time intervals with same exposure settings. Kymographs were generated from time-lapse VA-TIRFM movies using Fiji (ImageJ) v.2.0. software. WDL4-labeled cortical microtubule filaments with clearly resolved punctate signals were selected for analysis. For each selected event, a short linear ROI was drawn perpendicular to the WDL4-labeled filament at the position of the punctate signal. The same ROI was applied to both fluorescence channels using the ROI Manager, and kymographs were generated for each channel using the Multi Kymograph plugin. For intensity profile analysis, line profiles were extracted from the resulting kymographs at the same time point in both channels using the Plot Profile tool. The relative fluorescence intensity profiles of the two channels were then plotted to assess signal association at that time point.

### Statistical Analysis

Statistical analysis was performed using SPSS. Normality was assessed using the Shapiro-Wilk test. For datasets that did not show significant deviation from normality, Student’s t-test (two-group comparison) or one-way ANOVA followed by Tukey’s multiple comparisons test (multiple-group comparison) was used as needed. For datasets that significantly deviated from normality, Mann-Whitney U test (two-group comparison) or Kruskal-Wallis test followed by Dunn’s multiple comparisons test with Bonferroni-adjusted *P* values (multiple-group comparison) was used.

Data are presented either as mean ± SD or box plots, as indicated in the figure legends. Box plots show the median (center line), the 25th and 75th percentiles (box limits), and the minimum and maximum values (whiskers).

### Accession numbers

Sequence data of genes described in this article can be found in the Arabidopsis Genome Initiative under the following accession numbers: *WDL4* (At2g35880); *WDL5* (At4g32330); *PIN1* (At1g73590); *SYP121* (At3g11820); *VAMP721* (At1g04750); *KEULE* (At1g12360); *SNX1* (At5g06140); *CLASP* (At2g20190); *IAA1* (At4g14560); *SYP132* (At5g08080); *VAMP714* (At5g22360).
