# Supplementary material for: A MAP-SNARE interaction links cortical microtubules to the exocytic fusion machinery during auxin-dependent growth remodeling
Source: Sci Adv. 2026 Sep 2;12(36):eaef5793. doi: 10.1126/sciadv.aef5793 (PMC13537271; doi:10.1126/sciadv.aef5793)
Supplement: Supplementary file 1 [file sciadv.aef5793_sm.pdf]

Science Advances
AAAS

Supplementary Materials for

A MAP-SNARE interaction links cortical microtubules to the exocytic fusion machinery during auxin-dependent growth remodeling

Jia Deng *et al.*

Corresponding author: Tonglin Mao, maotl2005@cau.edu.cn; Xiangfeng Wang, wangxf2017@cau.edu.cn

Sci. Adv. **12**, eaef5793 (2026)
DOI: 10.1126/sciadv.aef5793

The PDF file includes:

Figs. S1 to S20
Legend for movie S1
Table S1

Other Supplementary Material for this manuscript includes the following:

Movie S1

Fig. S1.

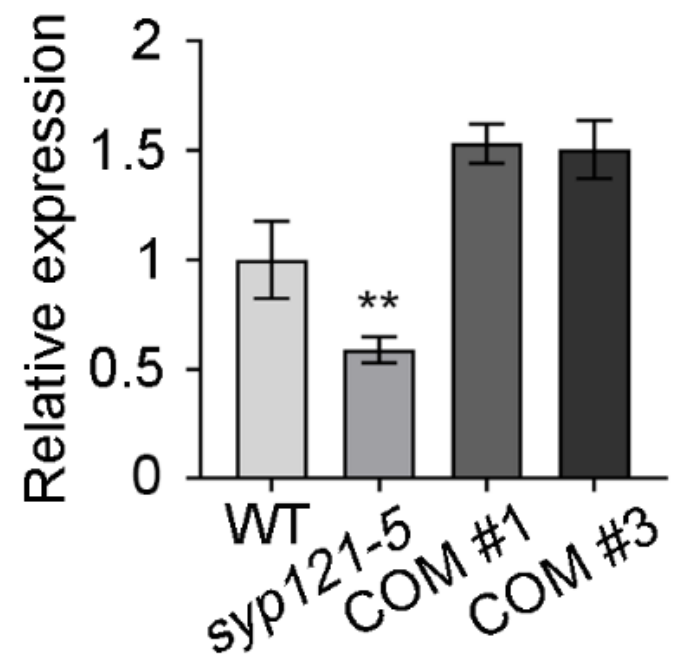

Supplemental Figure S1. Relative expression of *SYP121* in mutant and complementation plants.

The relative expression of *SYP121* in wild-type (WT), *syp121-5* mutant, and two independent *syp121-5 SYP121pro: GFP-SYP121* complementation lines (COM #1 and COM #3). The seedlings were grown in the dark for 3 d. Values represent mean ± SD for three individual experiment repeats. (Student's *t* test, $**P < 0.01$).

Fig. S2.

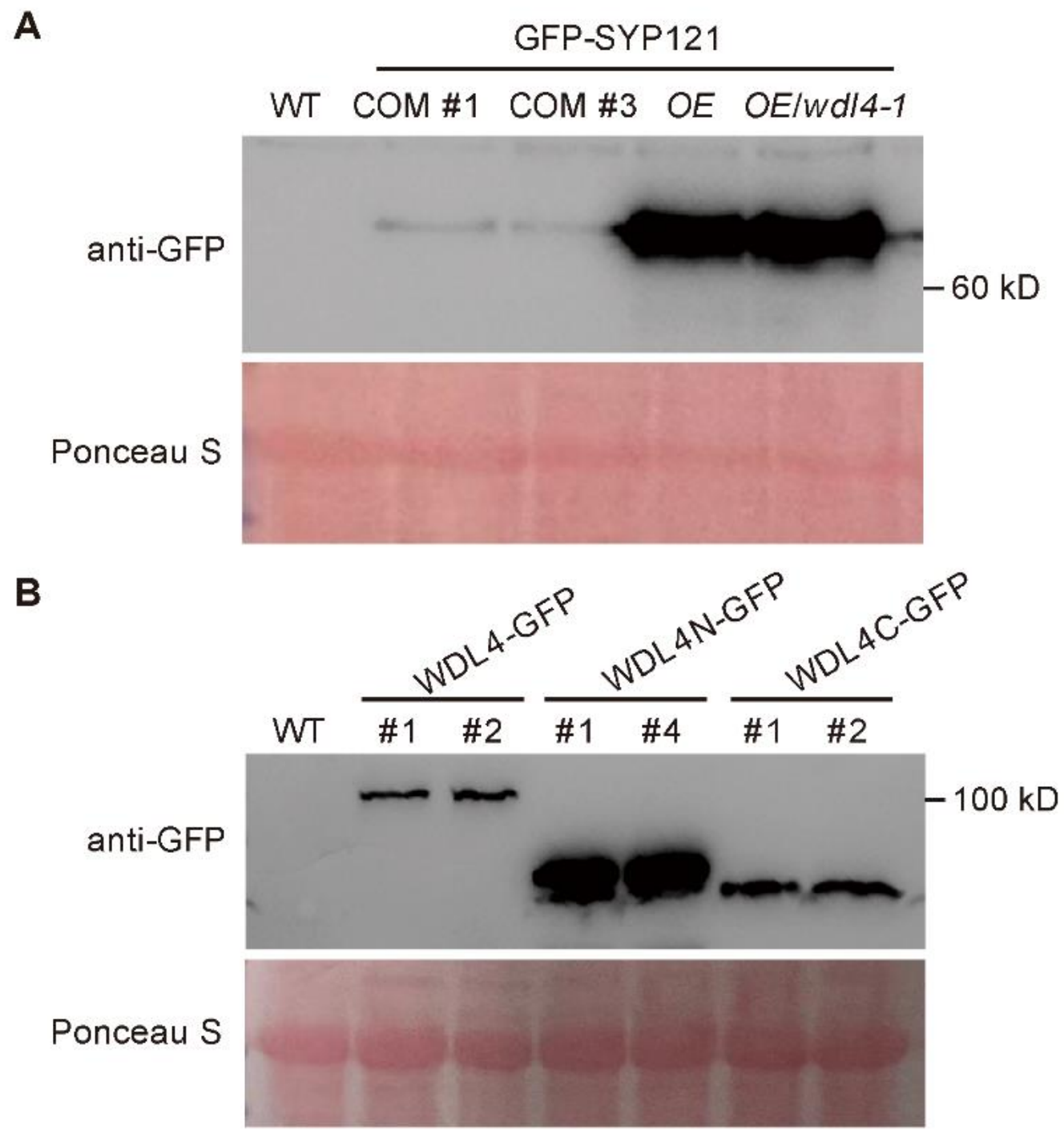

Supplemental Figure S2. Protein-level validation of overexpression and complementation lines.

(A) Immunoblot analysis of GFP-SYP121 protein accumulation in the indicated *SYP121* overexpression (OE) and complementation lines (COM) using an anti-GFP antibody. **(B)** Immunoblot analysis of WDL4/WDL4N/WDL4C-GFP protein accumulation using an anti-GFP antibody. WT seedlings were included as negative controls. Ponceau S was used as a loading control.

Fig. S3.

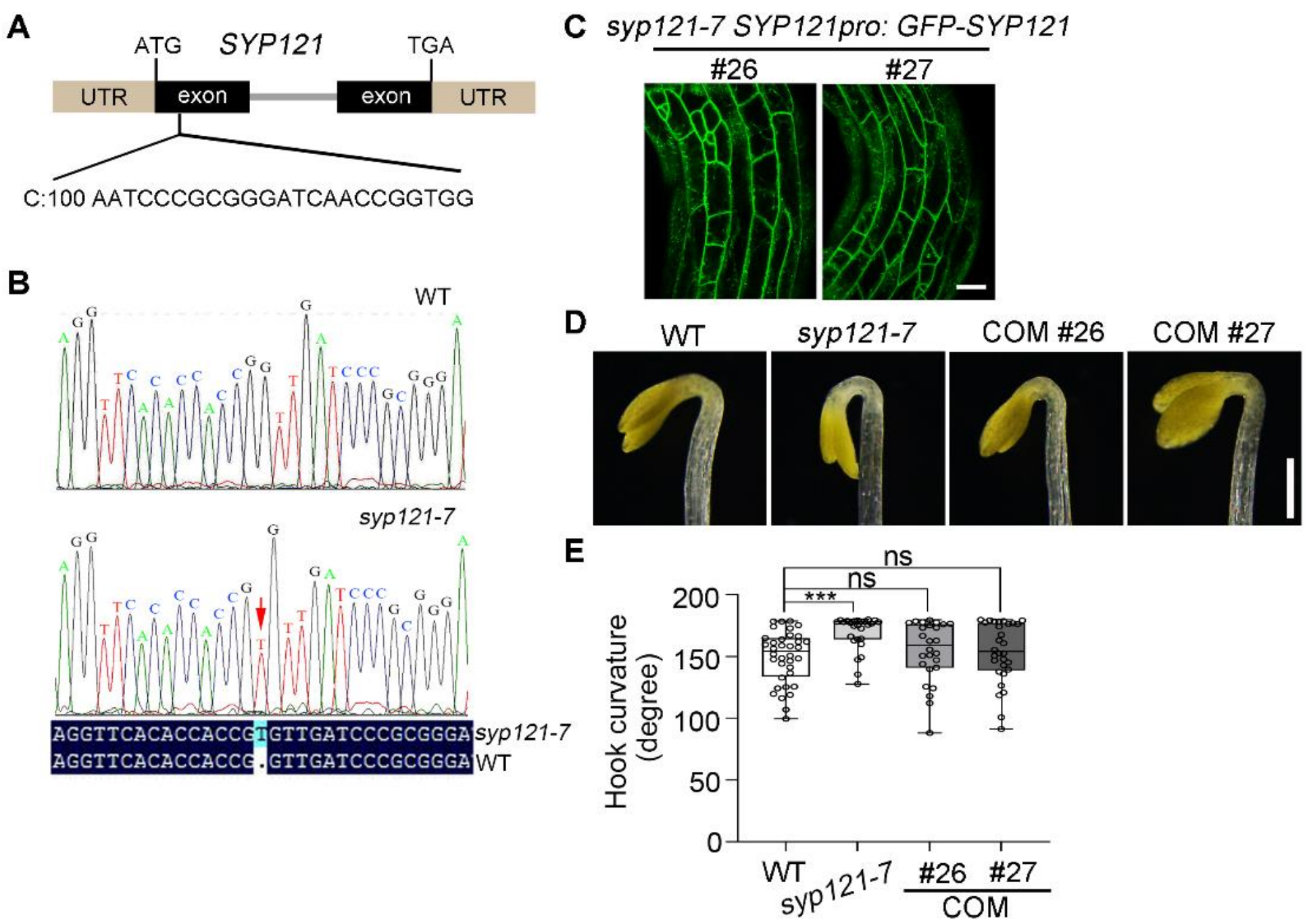

Supplemental Figure S3. The phenotype of *syp121-7* is complemented by the expression of *SYP121pro: GFP-SYP121*.

(A) Diagram showed the target sites of CRISPR/Cas9 system. **(B)** Sequencing results showed the mutation in *syp121-7* mutant generated by CRISPR/Cas9 technology. **(C)** Confocal images of apical hook epidermal cells from dark-grown 3-d-old *SYP121pro: GFP-SYP121* transgenic plants. Two independent lines were analyzed. Scale bar = 20 μm. **(D)** and **(E)** Representative images and quantitative analysis of the hook curvature in different seedlings. Seedlings were germinated and grown vertically in the dark for 72 h before imaging. Scale bar = 0.5 mm. Data are shown as box plots with individual data points (n ≥ 26 seedlings). Statistical significance was determined by Mann-Whitney U test (***$P < 0.001$). Experiments were repeated three times independently with similar results.

Fig. S4.

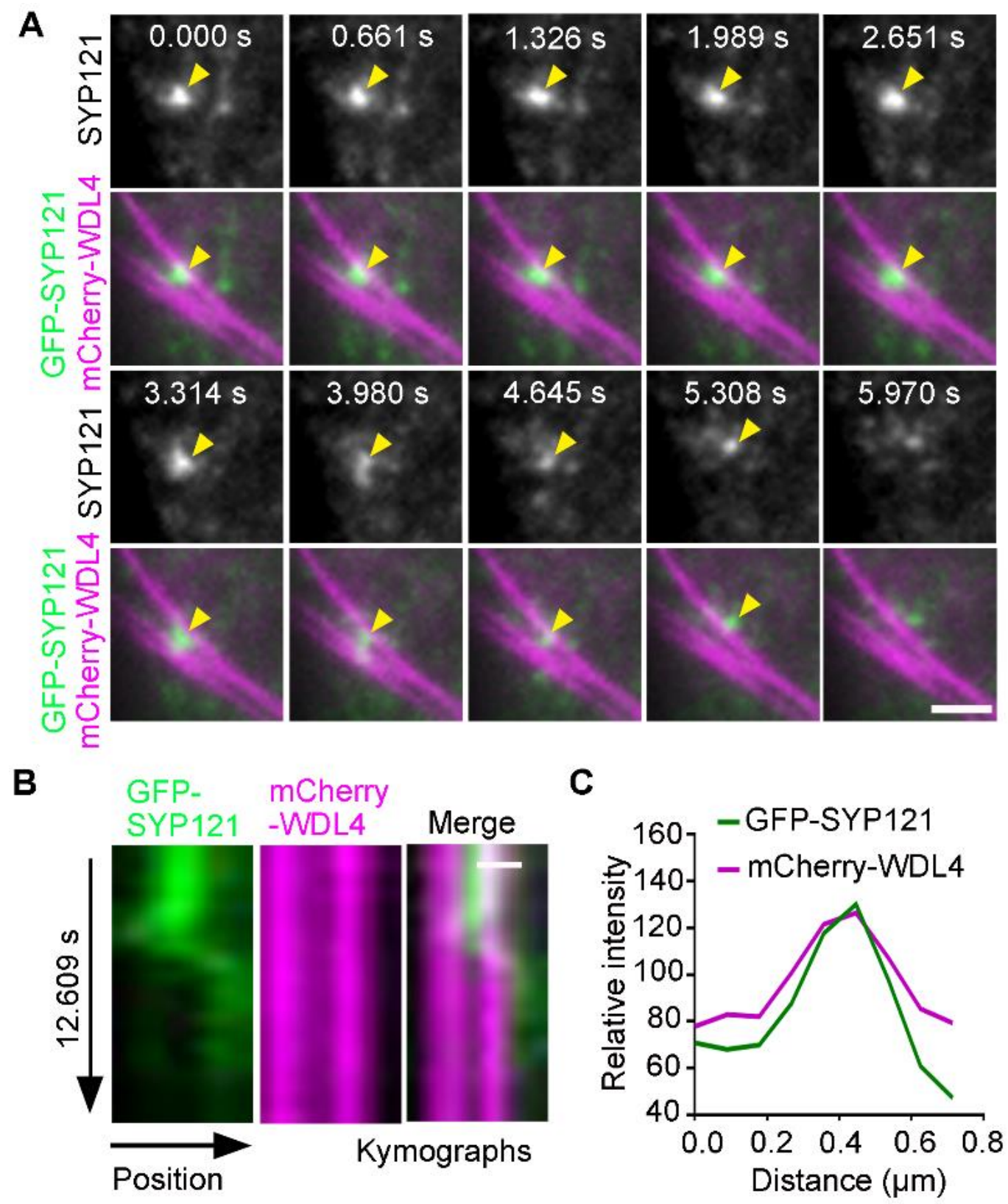

Supplemental Figure S4. SYP121 partially associates with WDL4-labeled microtubules in vivo.

(A) VA-TIRF microscopy imaging showed that PM-localized GFP-SYP121 (green) was associated with mCherry-WDL4-labeled filamentous structures (magenta) for a certain time in stable transgenic Arabidopsis plants. mCherry-WDL4 was driven by *Super* promoter and GFP-SYP121 was driven by *35S* promoter. Arrowheads indicate SYP121 punctate signal. Scale bar = 1 μm. **(B)** Representative kymograph showing the association of SYP121 (green) with WDL4 (magenta). **(C)** Plot of the line scan drawn in **(B)** showing the correlation between the spatial localization of mCherry-WDL4 and GFP-SYP121.

Fig. S5.

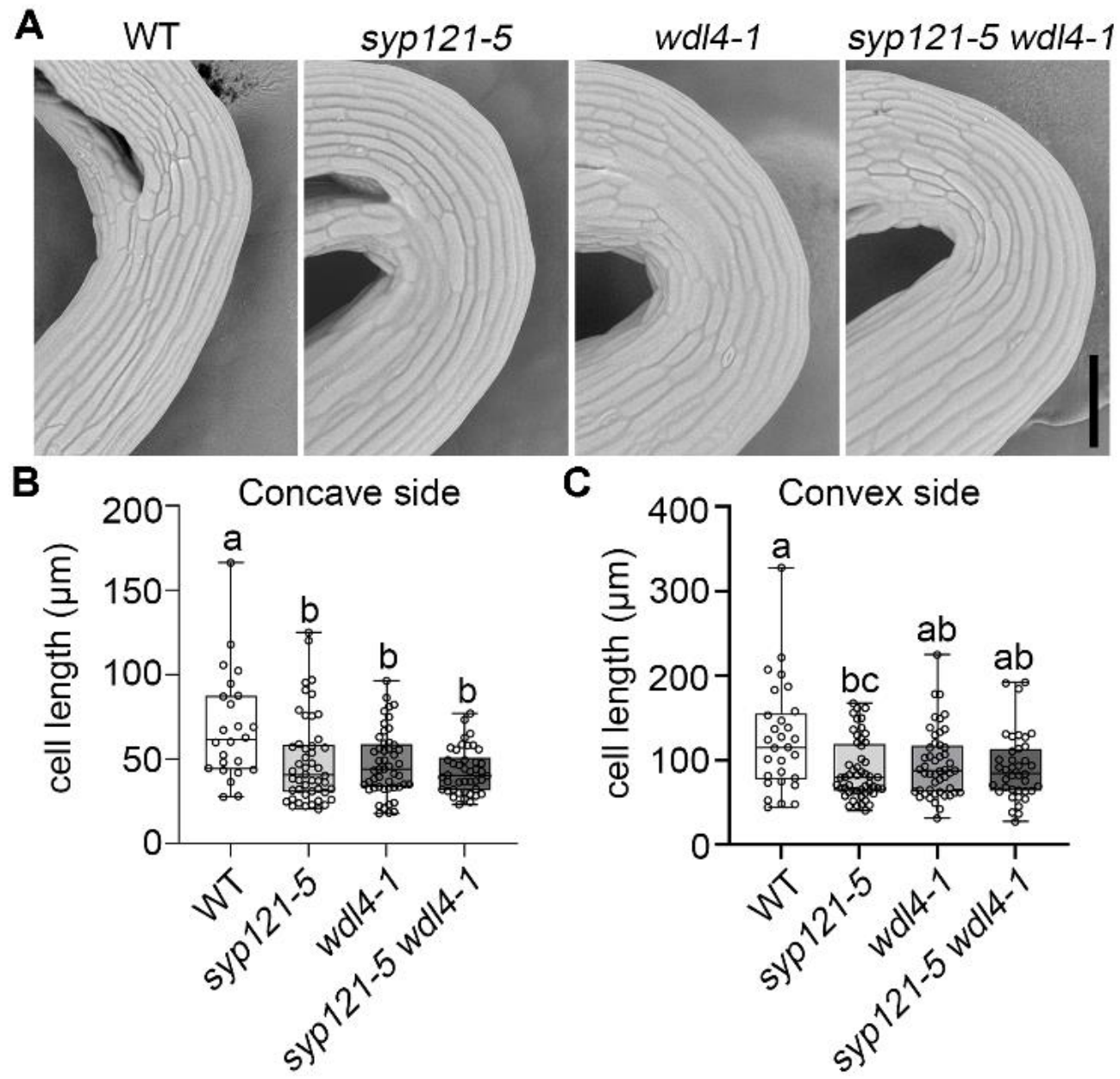

Supplemental Figure S5. Cell elongation defects on the concave side of the apical hook.

(A) Representative images using desktop scanning electron microscopy showing epidermal cell length on the concave (inner) and convex (outer) sides of the apical hook in 3-day-old dark-grown seedlings. Scale bar =100 µm. **(B)** and **(C)** Quantification of epidermal cell length on the concave and convex side. Data are shown as box plots with individual data points ($n \geq 24$). Statistical significance was determined by Kruskal-Wallis test followed by Dunn's multiple comparisons test with Bonferroni-adjusted *P* values. Different letters indicate significant differences at $P < 0.05$.

Fig. S6.

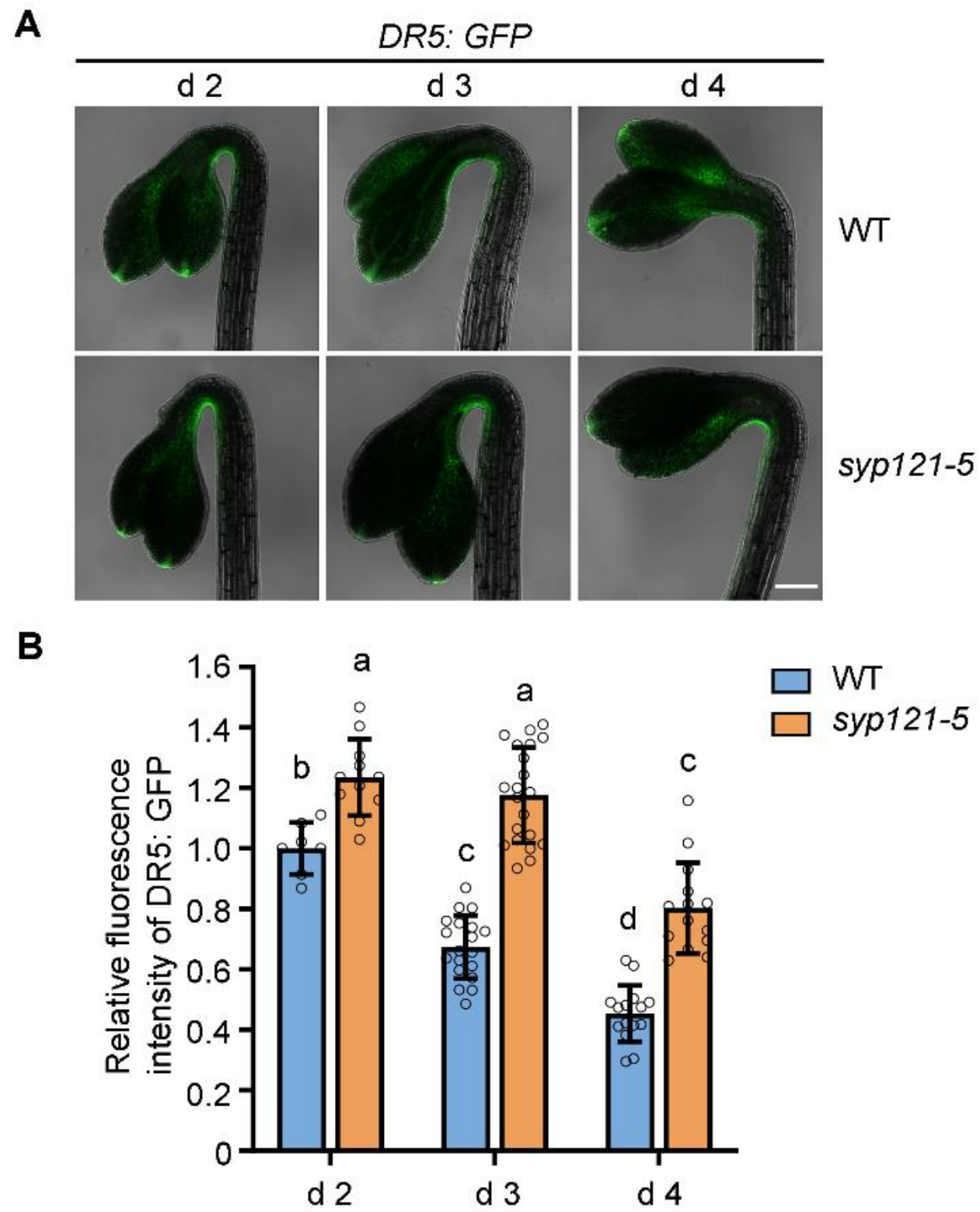

Supplemental Figure S6. ***SYP121*** **mutation alters auxin maxima at the concave side of the apical hook.**

(A) Representative images showed the expression pattern of *DR5: GFP* in the hook region of dark-grown WT and *syp121-5* mutants at different time points. Scale bar = 0.2 mm. **(B)** Quantitative analysis showed the relative intensity of DR5: GFP signals on the concave side of the hook region in WT and *syp121-5* seedlings. Seedlings were grown in the dark for 2-4 d. Values represent mean ± SD (n ≥ 7 seedlings). Different letters indicate significant differences (one-way ANOVA followed by Tukey's multiple comparisons test, $P < 0.05$).

Fig. S7.

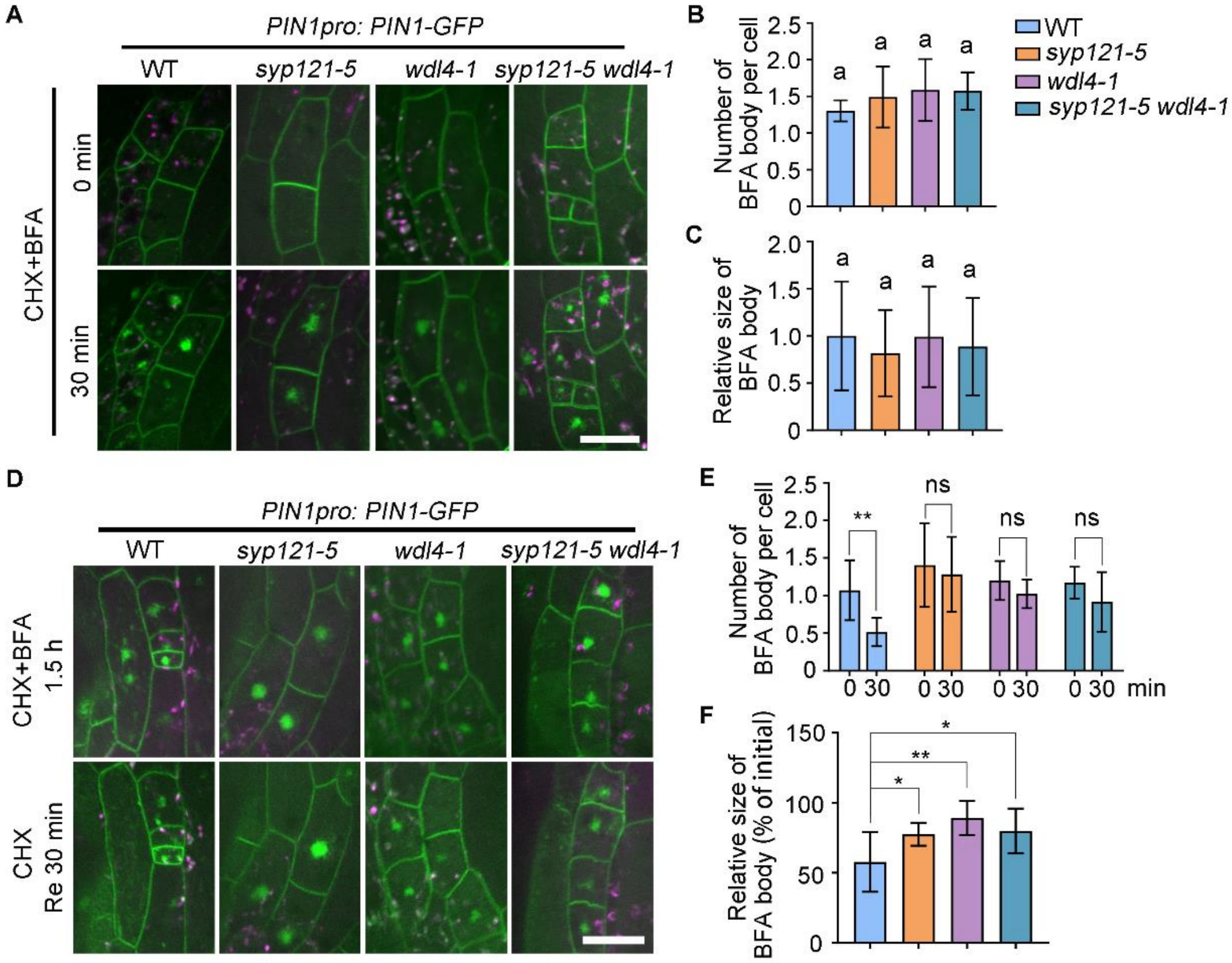

Supplemental Figure S7. WDL4 and SYP121 participate in PIN1 recycling during light-induced hook opening.

(A) Seedlings expressing *PIN1pro: PIN1-GFP* were germinated vertically and grown in the dark for 42 h, and transferred to white light illumination with CHX pretreatment (50 μM) for 60 min and then BFA (100 μM) in presence of CHX (50 μM) for another 30 min. The autofluorescence signals was shown in magenta. Scale bar = 20 μm. **(B)** and **(C)** Number of PIN1-GFP-labeled BFA bodies per cell and their relative size. The values represent the means ± SD (Kruskal-Wallis test followed by Dunn's multiple comparisons test with Bonferroni-adjusted *P* values). **(D)** Seedlings were pre-treated with CHX for 30 min, followed by CHX+BFA treatment for 1.5 h and BFA washout for 30 min while maintaining CHX. Scale bar = 20 μm. **(E)** and **(F)** Number of PIN1-GFP-labeled BFA bodies per cell and their relative size (% of initial). The values represent the means ± SD (Student's *t* test or Mann-Whitney U test, *$P < 0.05$, **$P < 0.01$).

Fig. S8.

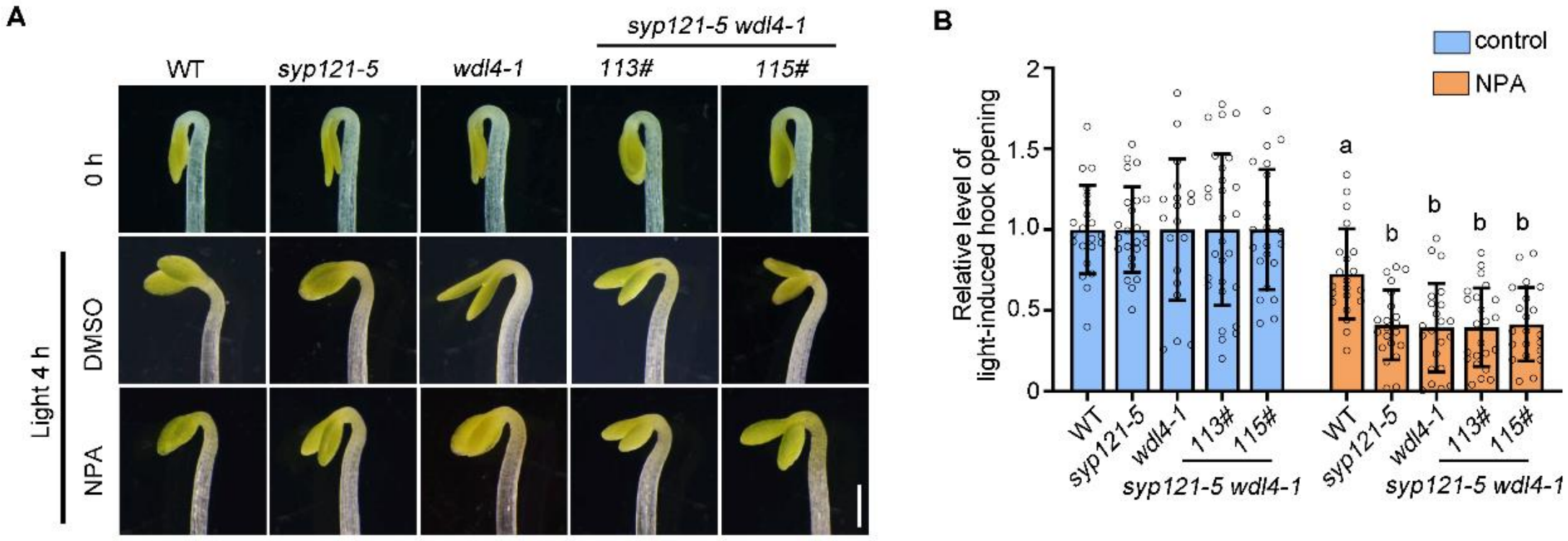

Supplemental Figure S8. NPA sensitivity during light-induced hook opening.

(A) Representative images of WT, *syp121-5*, *wdl4-1,* and *syp121-5 wdl4-1* seedlings during light-induced hook opening after DMSO or NPA treatment. Scale bar = 0.5 mm. **(B)** Quantification of relative hook opening under NPA treatment. Relative hook opening was calculated by normalizing the hook opening angle under NPA treatment to that under DMSO treatment for each genotype. Lower values indicate stronger sensitivity to auxin efflux inhibition. Data are shown as mean ± SD ($n \geq 21$). Different letters indicate significant differences (one-way ANOVA followed by Tukey's multiple comparison test, $P < 0.05$).

Fig. S9.

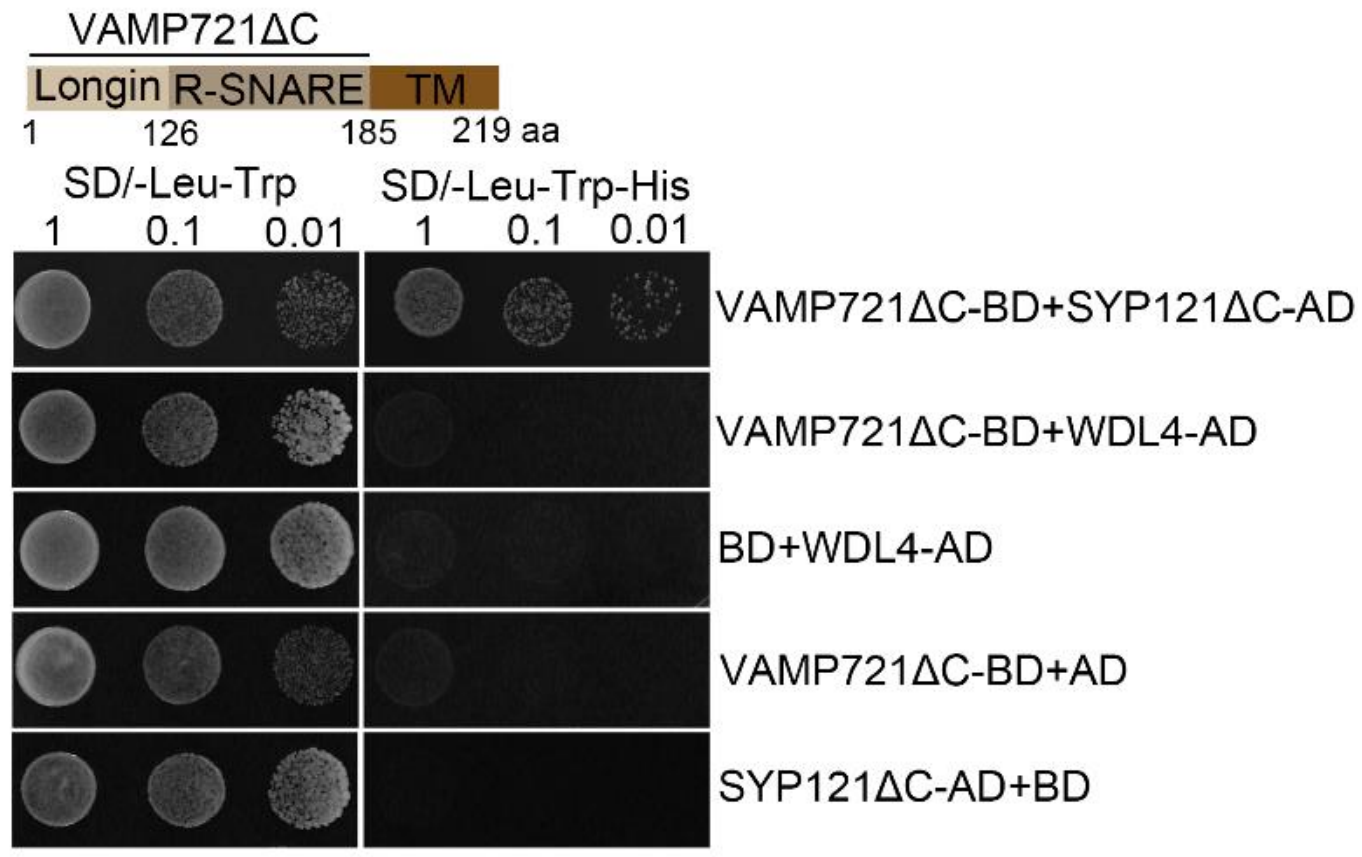

Supplemental Figure S9. WDL4 does not interact with VAMP721 in Yeast two-hybrid assay.

The diagram showed three distinct domains of VAMP721: the longin domain (residues 1-126), the R-SNARE domain (residues 127-185) and C-terminal transmembrane domain TM (residues 186-219). The cytosolic domain of VAMP721 (VAMP721ΔC) was used for the yeast two-hybrid assay. The combination of VAMP721ΔC-BD and SYP121ΔC-AD was used as positive control. Empty vectors were used as negative controls. 5 mM 3-AT was used to inhibit autoactivation.

Fig. S10.

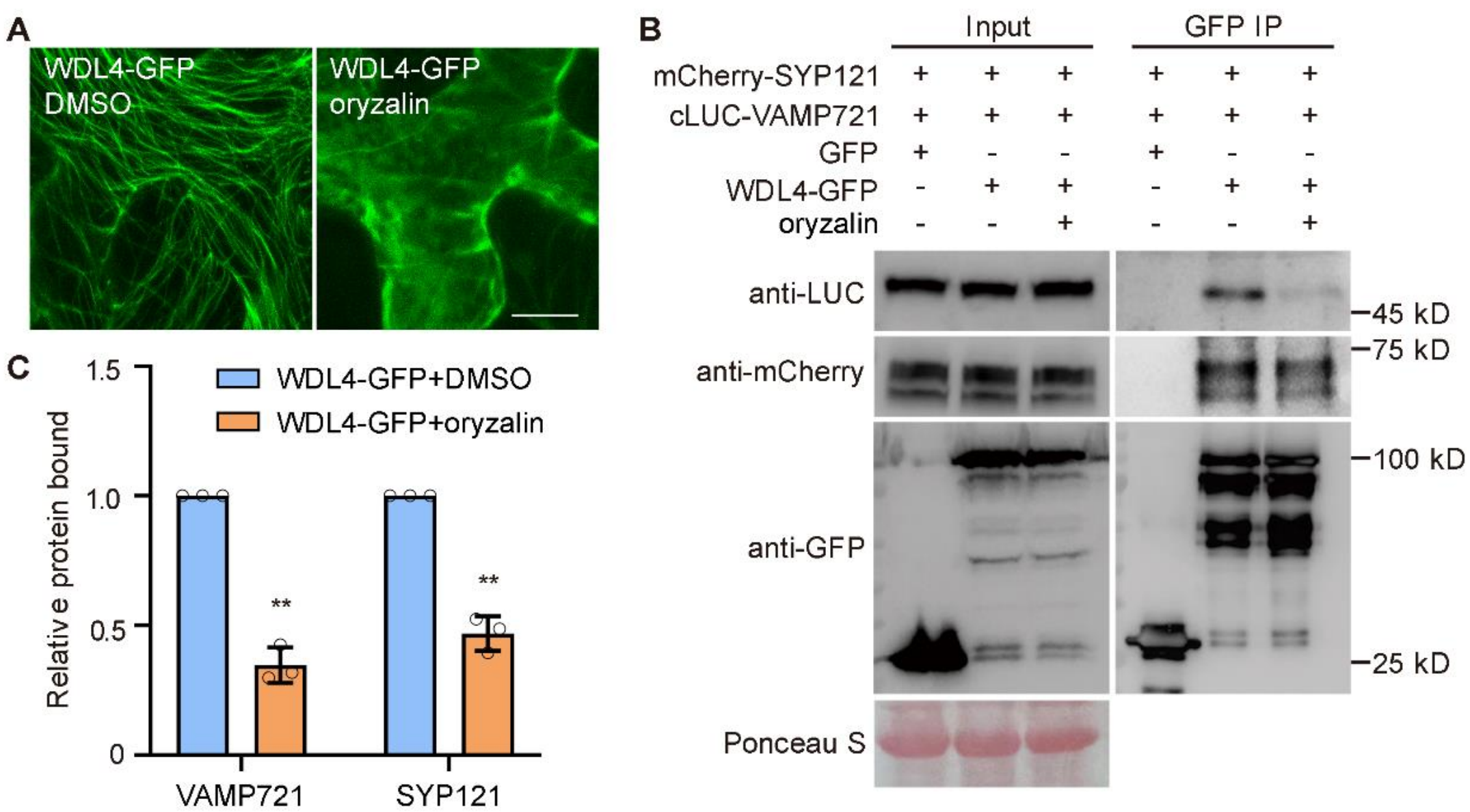

Supplemental Figure S10. Oryzalin treatment compromises the formation or stability of WDL4-SYP121-VAMP721 complex.

(A) Representative confocal images showing the effect of oryzalin treatment on WDL4-GFP-labeled cortical microtubule-associated structures in *N. benthamiana* leaf epidermal cells. Scale bar = 20 μm. **(B)** Co-IP assay showing the effect of oryzalin treatment on the formation of WDL4-SYP121-VAMP721 complex in *N. benthamiana* leaves. WDL4-GFP or free GFP was immunoprecipitated, and SYP121 and VAMP721 were co-immunoprecipitated and detected by immunoblotting. **(C)** Quantification of relative protein bound with or without oryzalin treatment. Data represent mean ± SD from three independent experiments with similar results (Student's *t* test, $^{**}P < 0.01$).

Fig. S11.

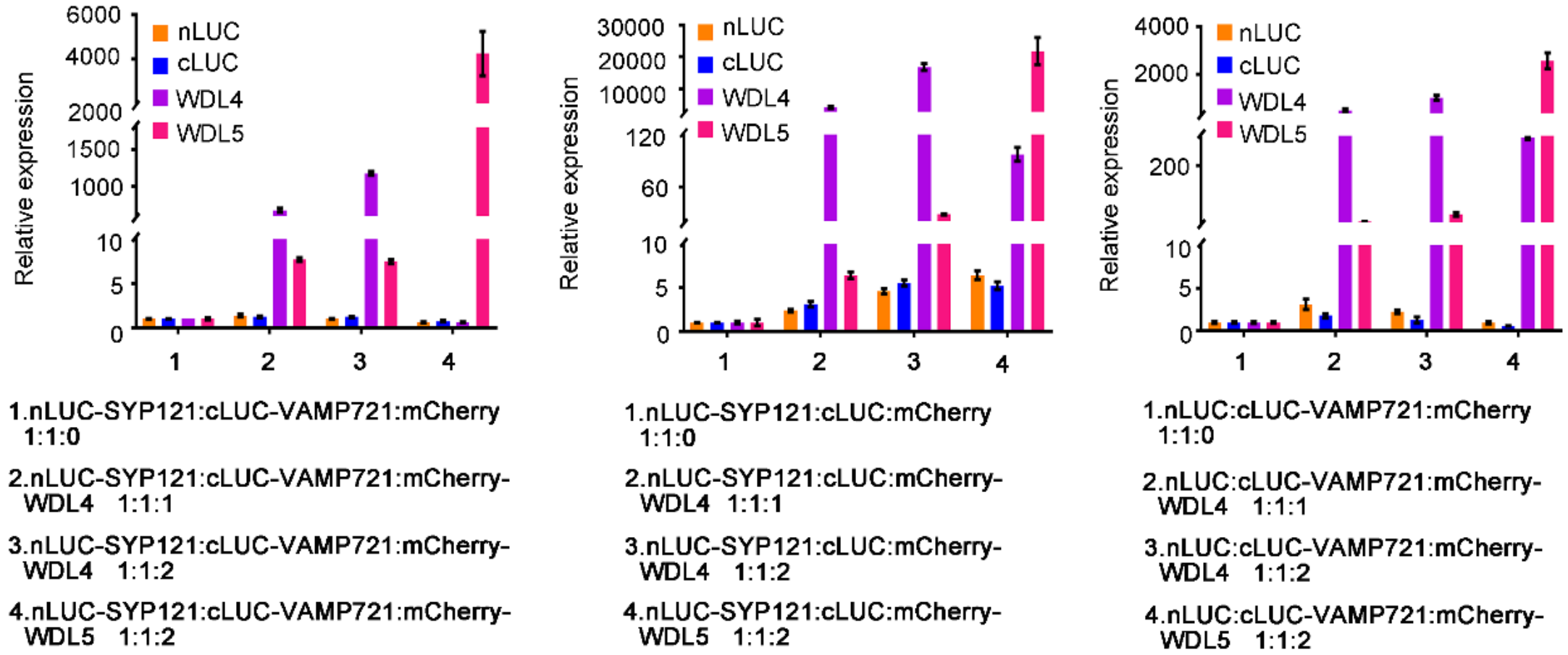

Supplemental Figure S11. Relative expression of *nLUC, cLUC, WDL4* and *WDL5* in the LCI assays.

Values represent mean ± SD for three individual experiment repeats.

Fig. S12.

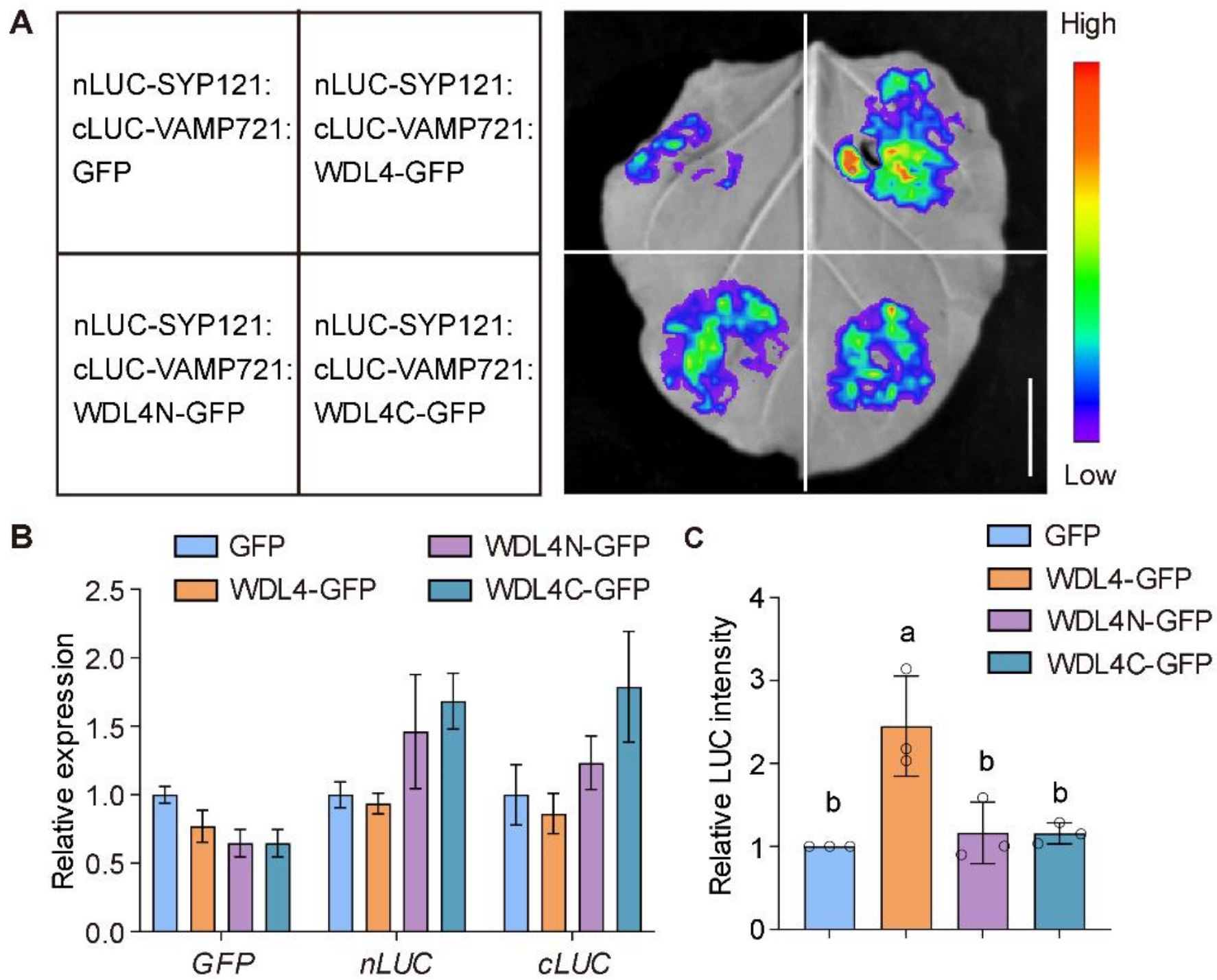

Supplemental Figure S12. Full-length WDL4 is required to enhance the interaction between SYP121 and VAMP721.

(A) Luciferase complementation imaging (LCI) assay showing the effect of full-length WDL4, WDL4N, and WDL4C on the interaction between SYP121 and VAMP721 in *N. benthamiana* leaves. Scale bar = 1 cm. **(B)** qRT-PCR analysis showing the relative expression levels of the indicated constructs in different groups. **(C)** Quantification of relative LUC intensity in the indicated groups. Data are shown as mean ± SD from three independent biological replicates. Different letters indicate significant differences (one-way ANOVA followed by Tukey's multiple comparison test, $P < 0.05$).

Fig. S13.

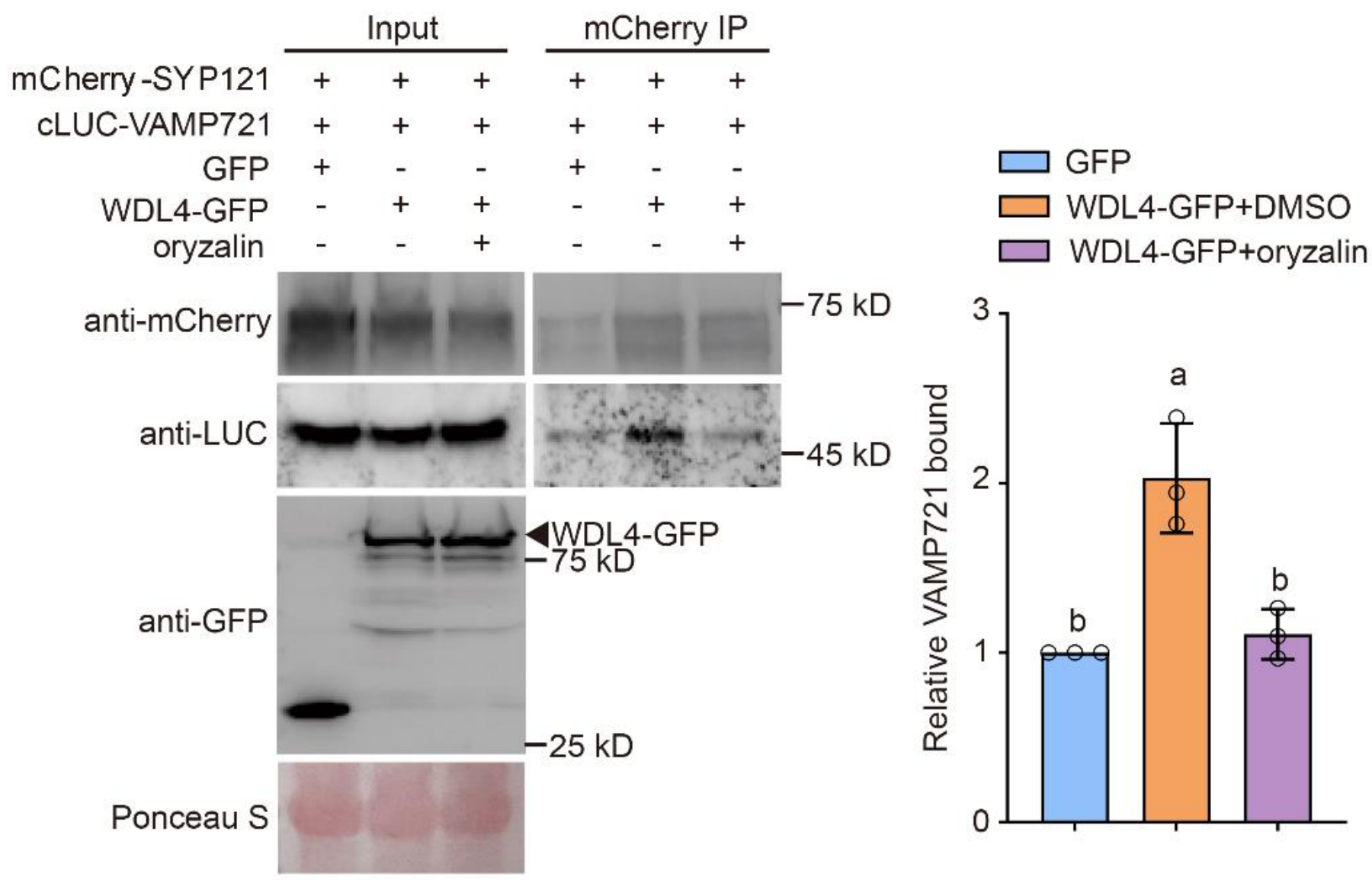

Supplemental Figure S13. Oryzalin treatment reduces WDL4-mediated enhancement of the SYP121-VAMP721 interaction.

Co-IP assay in *N. benthamiana* leaves. mCherry-SYP121 was used as the bait for immunoprecipitation. VAMP721 was co-immunoprecipitated and detected by immunoblot. The relative amount of VAMP721 binding to SYP121 was quantified. Data represent mean ± SD from three independent experiments with similar results. Different letters indicate significant differences (one-way ANOVA followed by Tukey's multiple comparison test, $P < 0.05$).

Fig. S14.

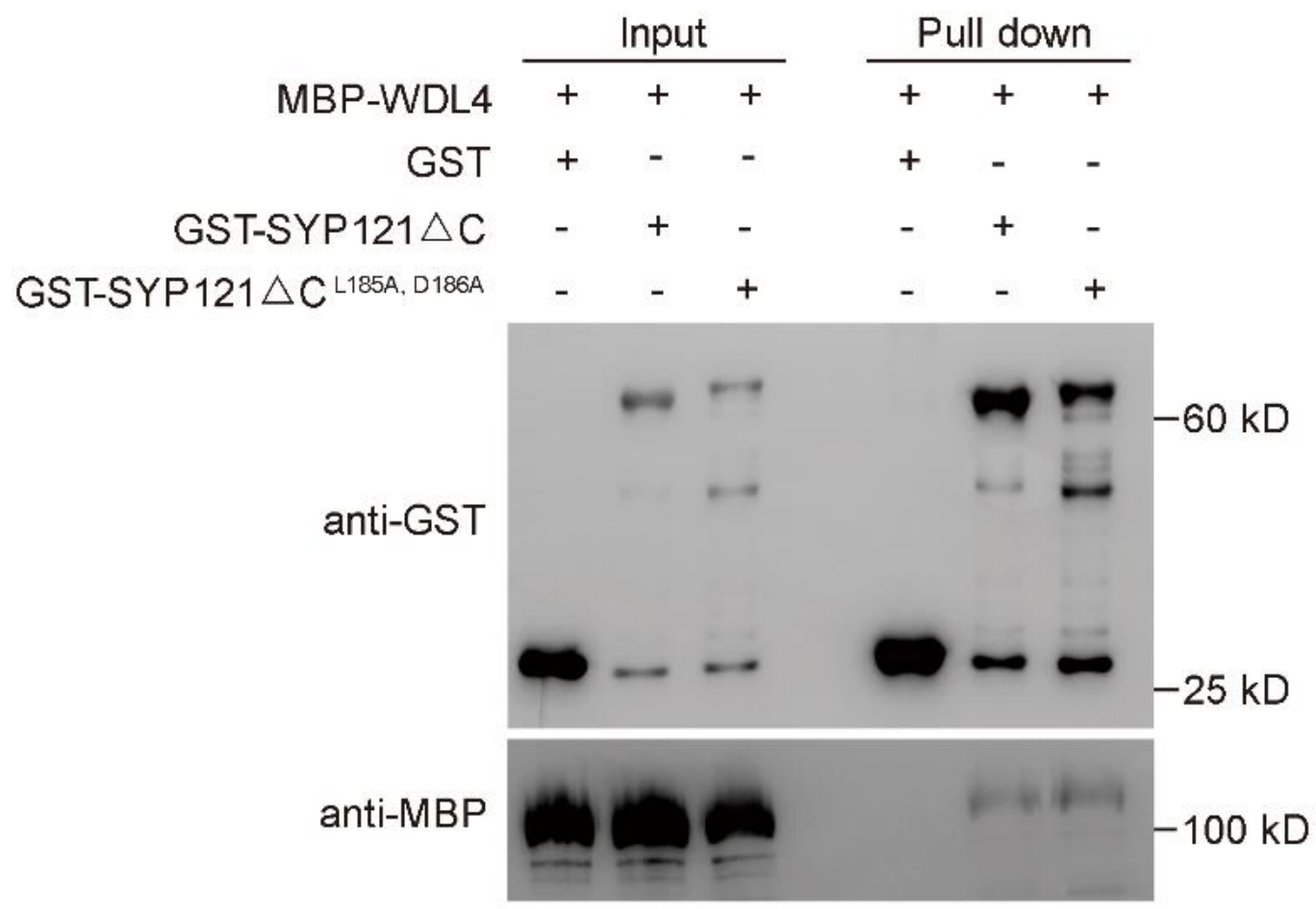

Supplemental Figure S14. SYP121ΔC$^{L185A,\ D186A}$ mutation did not impair its interaction with WDL4.

In vitro pull-down assay showed that the SYP121ΔC$^{L185A,\ D186A}$ mutation did not impair its interaction with WDL4. The experiment was repeated three times independently with similar results.

Fig. S15.

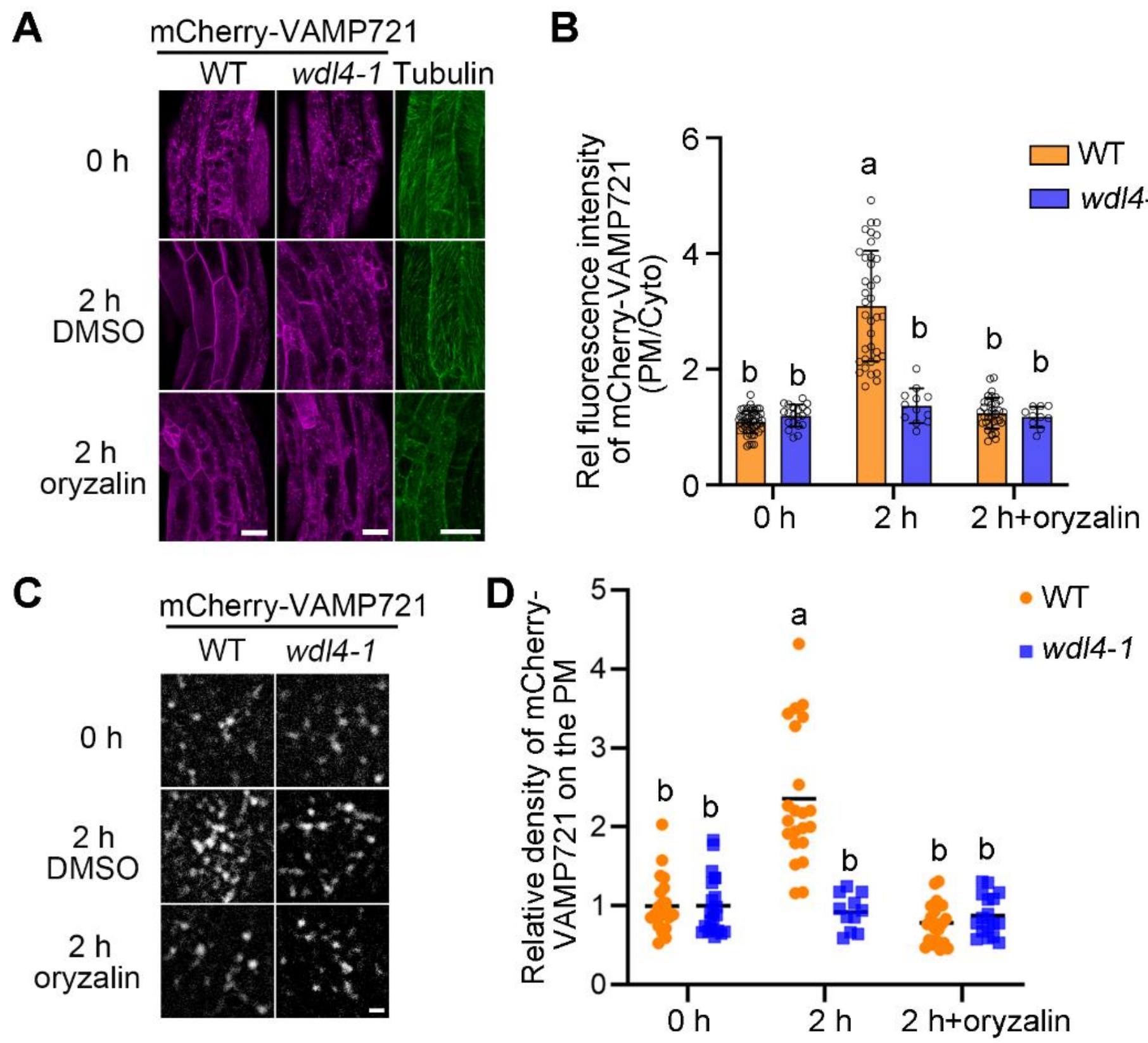

Supplemental Figure S15. Oryzalin treatment reduces the PM accumulation of VAMP721 during apical hook opening.

(A) Representative confocal images showing mCherry-VAMP721 localization in the apical hook region of WT and *wdl4-1* seedlings after light illumination with or without oryzalin treatment. Scale bar = 20 μm. **(B)** Quantification of the plasma membrane/cytoplasm (PM/Cyto) ratio of mCherry-VAMP721. The values represent mean ± SD (n ≥ 10 cells from at least 4 seedlings, one-way ANOVA followed by Tukey's multiple comparison test, $P < 0.05$). **(C)** Representative VA-TIRFM images showing PM-associated mCherry-VAMP721 foci after light illumination with or without oryzalin treatment. Scale bar = 1 μm. **(D)** Quantification of the relative density of mCherry-VAMP721 foci on the PM. The values represent mean ± SD (n ≥ 11 cells from at least 3 seedlings). Different letters indicate significant differences (Kruskal-Wallis test followed by Dunn's multiple comparisons test with Bonferroni-adjusted *P* values, $P < 0.05$).

Fig. S16.

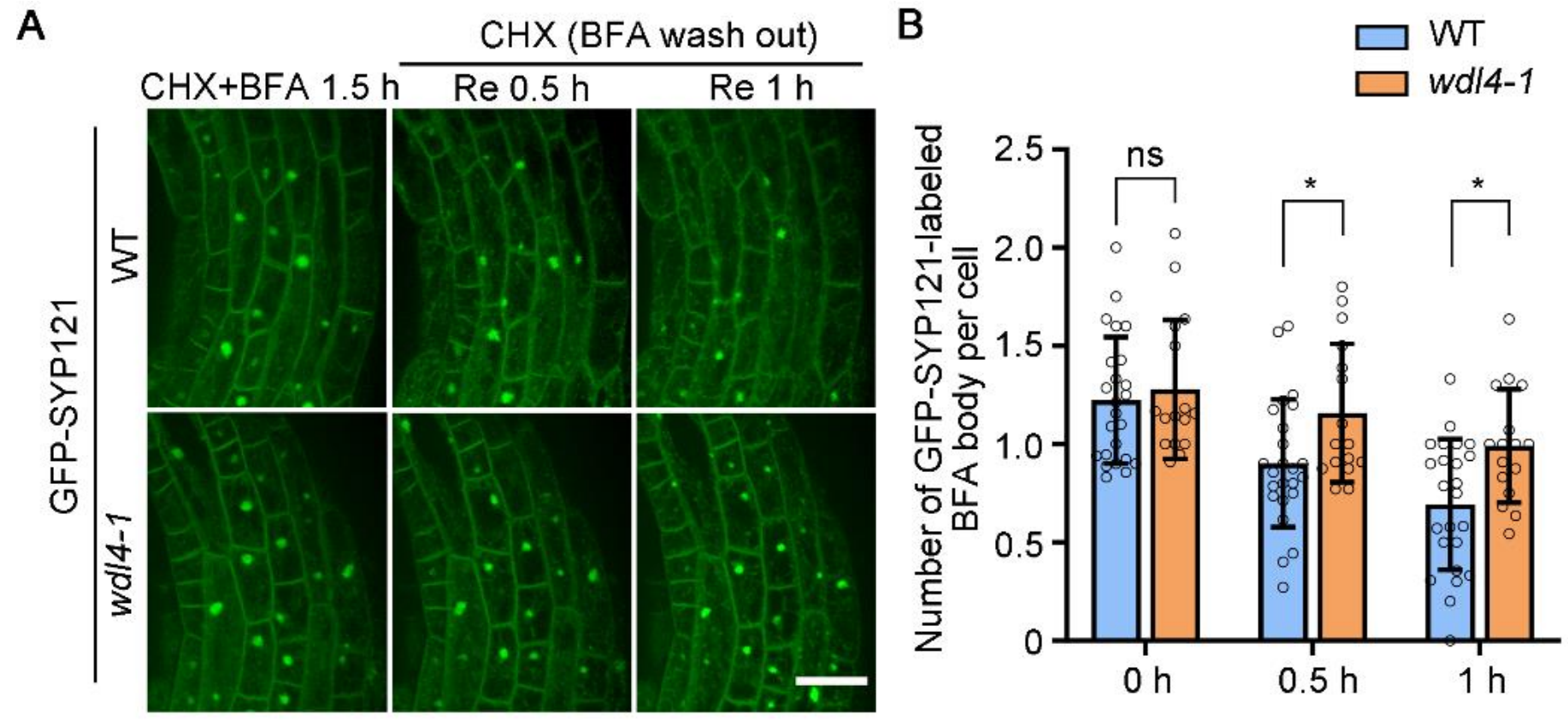

Supplemental Figure S16. WDL4 participates in SYP121 recycling during light-induced hook opening.

(A) Seedlings expressing *35S: GFP-SYP121* in WT and *wdl4-1* mutant background were germinated vertically and grown in the dark for 42 h, and transferred to white light illumination with CHX pretreatment (50 µM) for 30 min, followed by CHX+BFA treatment for 1.5 h and BFA washout for 1 h while maintaining CHX. Scale bar = 20 µm. **(B)** Number of GFP-SYP121-labeled BFA bodies per cell. The values represent the means ± SD (n ≥ 16 seedlings). Significant differences are determined by Mann-Whitney U test (*$P < 0.05$).

Fig. S17.

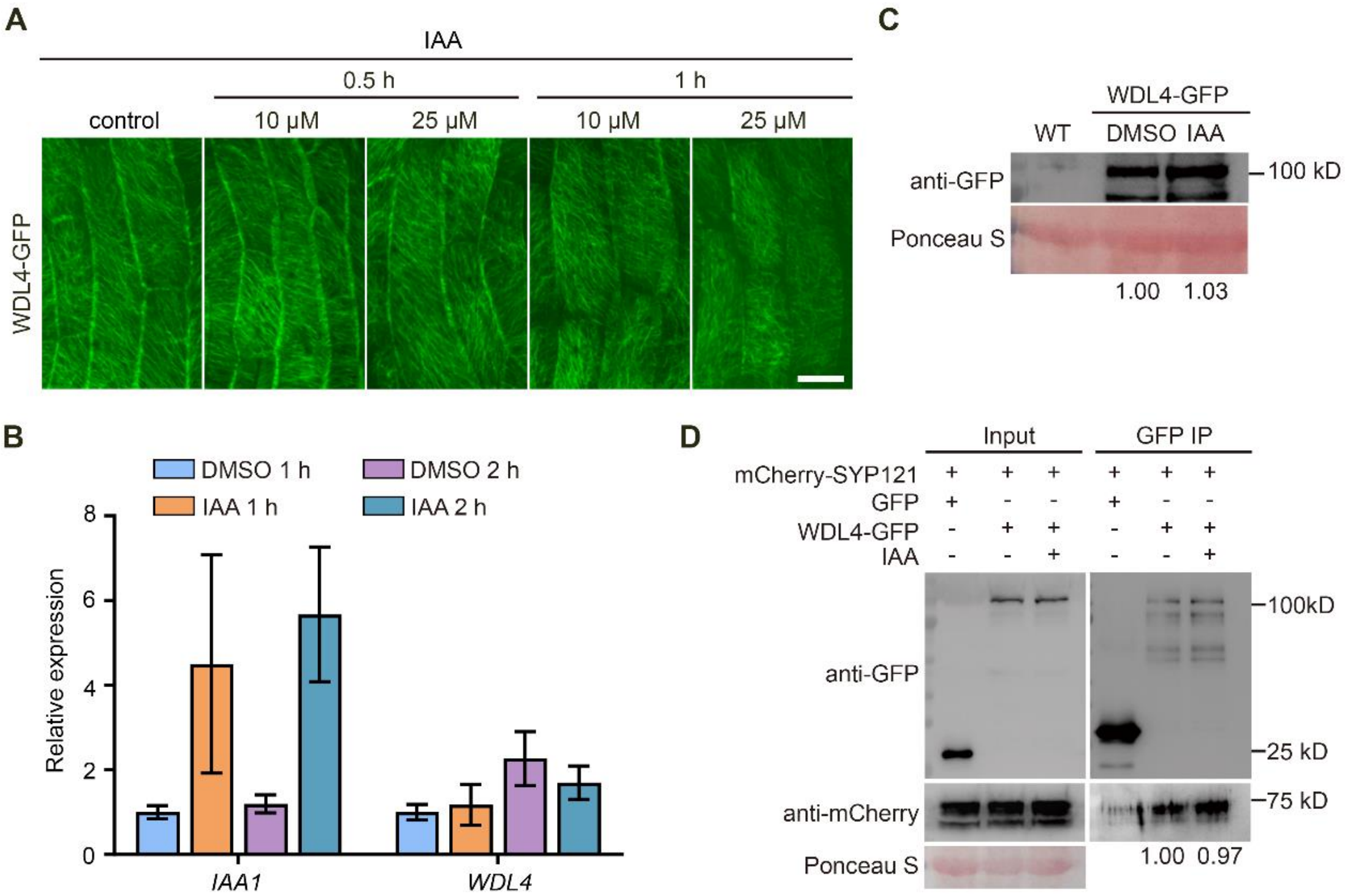

Supplemental Figure S17. Effect of IAA treatment on WDL4.

(A) Representative confocal images of WDL4-GFP localization in the apical hook region after mock or IAA treatment. The *WDL4-GFP* complementation seedlings were grown in the dark for 3 d, and treated with 25 µM IAA for indicated durations before confocal observation. Scale bar = 20 µm. **(B)** qRT-PCR analysis of *WDL4* transcript levels after DMSO or IAA treatment. Seedlings were grown in the dark for 3 d, and treated with 25 µM IAA for indicated durations. 1-2 mm of the apical hook regions were collected for RNA extraction. Values represent mean ± SD for three individual experiment repeats. **(C)** Immunoblot analysis of WDL4-GFP protein accumulation after DMSO or IAA treatment. The seedlings were grown in the dark for 3 d, and treated with 25 µM IAA for 1 h before protein extraction. 1-2 mm of the apical hook regions were collected. The experiments were repeated three times independently with similar results. Numbers below the blot indicate relative WDL4-GFP levels. **(D)** Co-IP assay showing the effect of IAA on the WDL4-SYP121 interaction in *N. benthamiana* leaves. The leaves were treated with 25 µM IAA for 1 h before protein extraction. The experiments are repeated three times independently with similar results. Numbers indicate relative co-immunoprecipitated SYP121 normalized to WDL4.

Fig. S18.

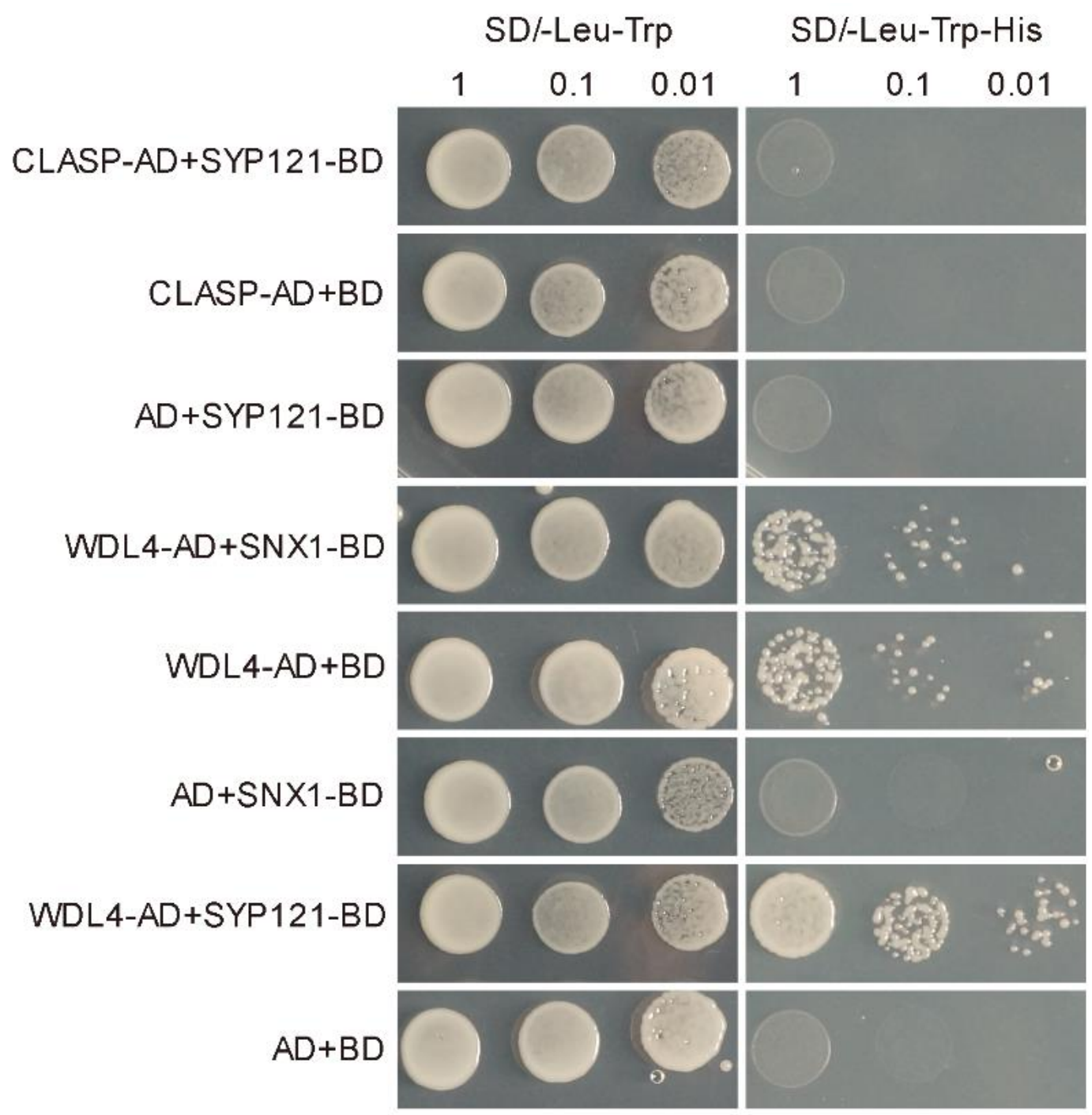

Supplemental Figure S18. Y2H assays between the WDL4-SYP121 and CLASP-SNX1 modules.

Yeast two-hybrid assay showed no detectable interaction between CLASP and SYP121 or between WDL4 and SNX1. Empty vectors were used as negative controls. WDL4 and SYP121 were used as positive control. 3 mM 3-AT was used to inhibit autoactivation.

Fig. S19.

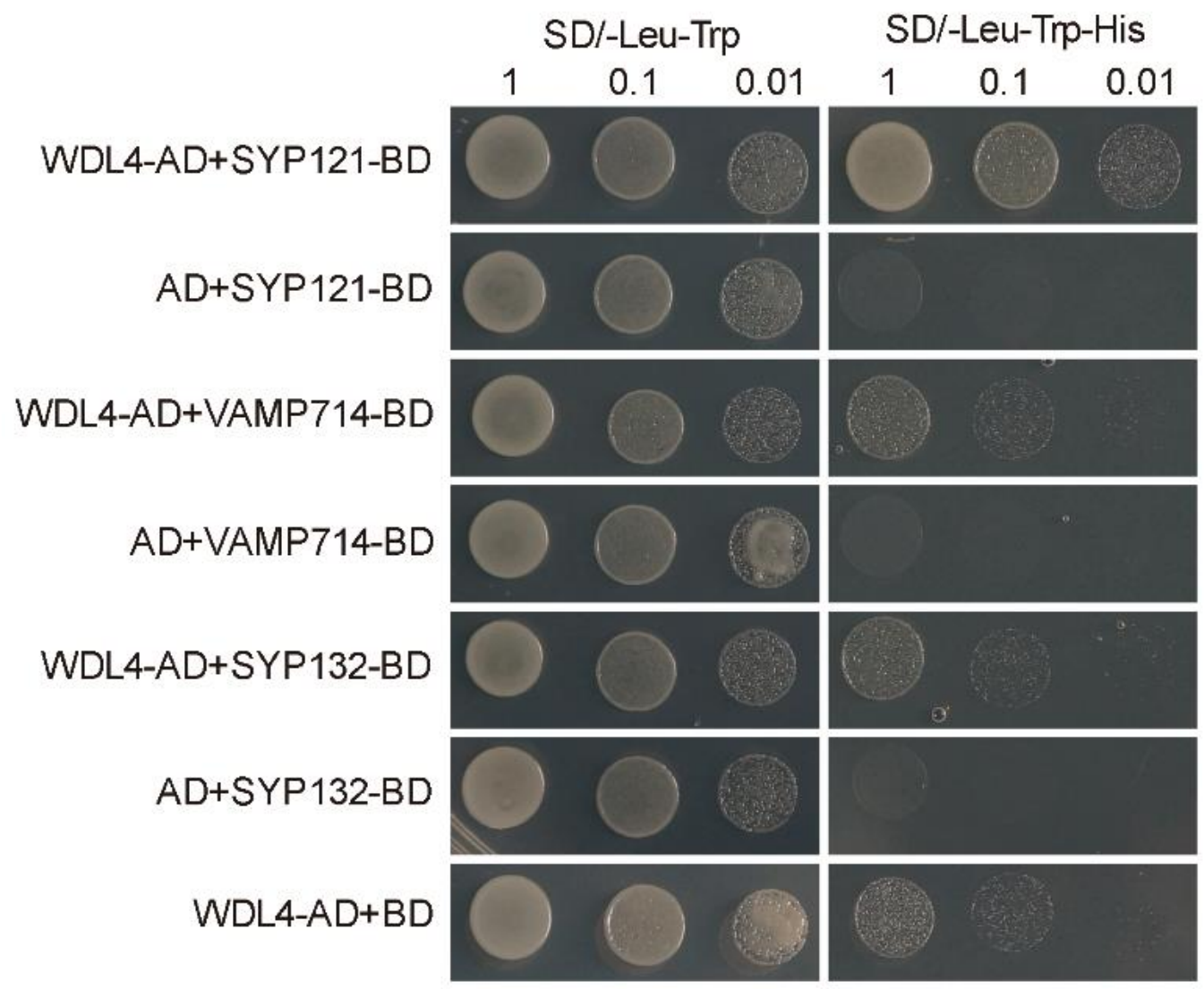

Supplemental Figure S19. Y2H assays between the WDL4 and other SNARE proteins.

Yeast two-hybrid assay showed no detectable interaction between WDL4 and VAMP714 and SYP132. Empty vectors were used as negative controls. WDL4 and SYP121 were used as positive control. 5 mM 3-AT was used to inhibit autoactivation.

Fig. S20.

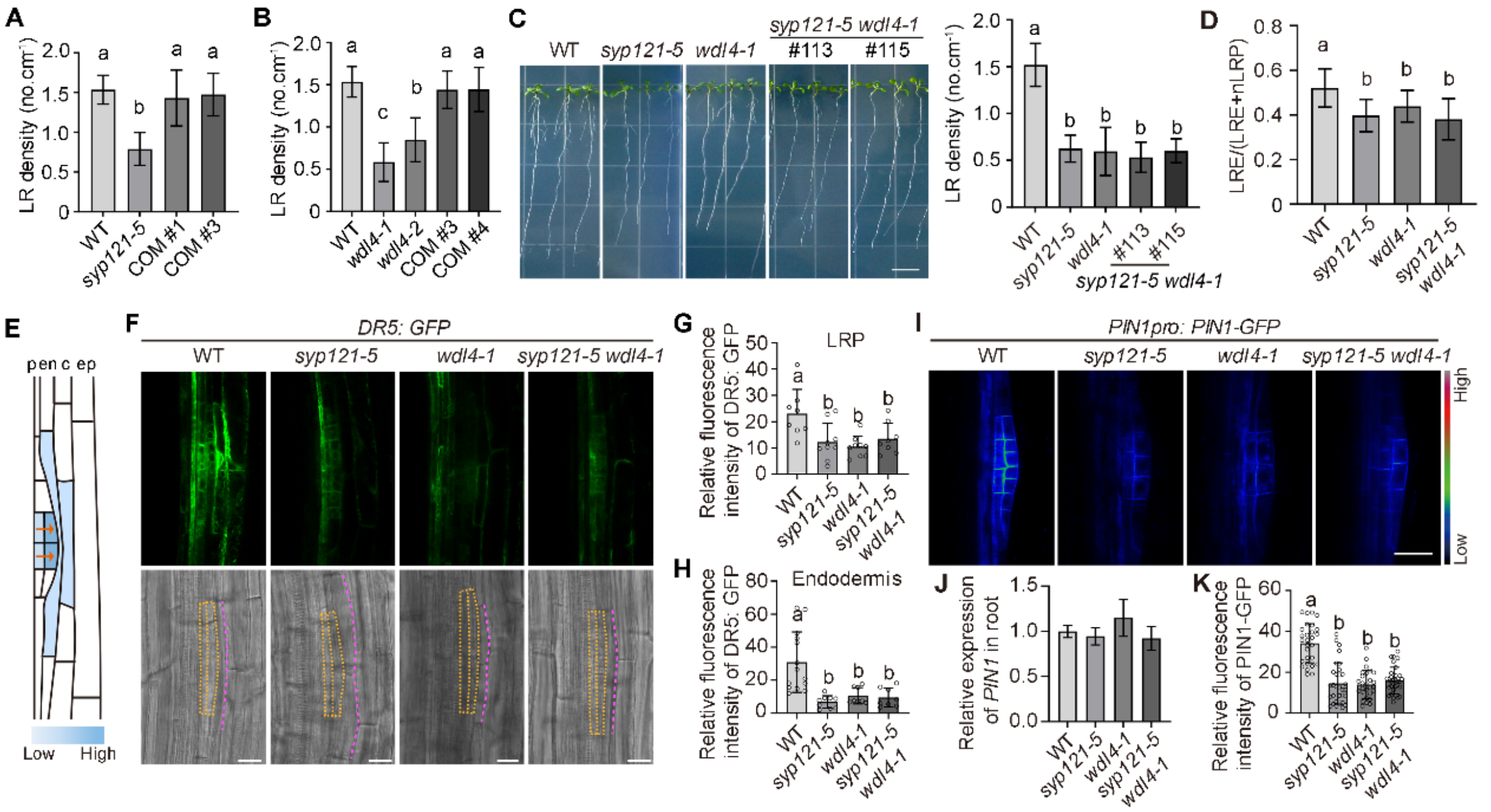

Supplemental Figure S20. The SYP121-WDL4 module contributes to lateral root emergence. (A) and **(B)** Quantification of lateral root (LR) density in *syp121-5* and its complementation lines, as well as *wdl4-1, wdl4-2,* and *wdl4-2* complementation lines. Seedlings were germinated and grown in the light for 10 d. Values represent mean ± SD (n ≥ 14 seedlings). **(C)** LR phenotype and LR density quantification of WT, *syp121-5, wdl4-1*, and two independent *syp121-5 wdl4-1* double-mutant lines. Seedlings were germinated and grown in the light for 10 d. Scale bar = 1 cm. Values represent mean ± SD (n ≥ 10 seedlings). **(D)** Ratio of emerged lateral roots (LRE) to total lateral root primordia (LRE + nLRP, where nLRP indicates non-emerged lateral root primordia). Values represent mean ± SD (n ≥ 15 seedlings). **(E)** Schematic view of auxin accumulation in stage II LRP and overlying cell layers. p: pericycle; en: endodermis; c: cortex; ep: epidermis. Arrows indicate PIN1-mediated auxin flow. Blue gradient indicates auxin distribution. **(F)** Representative images of DR5: GFP expression in LRP of 6-day-old light grown seedlings. Yellow dotted lines indicate the boundary of developing stage II LRP. Magenta dotted lines indicate the boundary of the overlying endodermis cells in the main root. Scale bar = 20 µm. **(G)** and **(H)** Quantification of relative intensity of DR5: GFP signals in LRP and overlying endodermis cells. Values represent mean ± SD (n ≥ 8 seedlings). **(I)** Representative images of PIN1-GFP subcellular distribution in LRP of the indicated genotypes expressing *PIN1pro: PIN1-GFP*. PIN1-GFP signals were shown

as pseudocolors (red-green-blue palette). Seedlings were germinated and grown in the light for 6 d. Scale bar = 20 μm. **(J)** Relative expression of *PIN1* in roots of the indicated genotypes expressing *PIN1pro: PIN1-GFP*. Seedlings were grown in the light for 6 d. Values represent mean ± SD for three independent experiment repeats. **(K)** Quantification of relative PIN1-GFP fluorescence intensity at the PM of primordia cells. Values represent mean ± SD ($n \geq 15$ seedlings). For **(A)**, **(C)**, **(H)** and **(K)**, different letters indicate significant differences by Kruskal-Wallis test followed by Dunn's multiple comparisons test with Bonferroni-adjusted *P* values ($P < 0.05$). For **(B)**, **(D)** and **(G)**, different letters indicate significant differences by one-way ANOVA followed by Tukey’s multiple comparison test ($P < 0.05$).

Movie S1.

Supplemental video S1. Time-lapse imaging shows the association between SYP121 and WDL4-labeled microtubules in vivo.

VA-TIRF microscopy imaging showed mCherry-SYP121 (magenta) associates with WDL4-labeled microtubules (green) for a certain duration in stable transgenic Arabidopsis plants. Both *WDL4-GFP* and *mCherry-SYP121* were driven by their native promoters. Scale bar = 5 μm.

Table S1.

Supplemental Table S1. List of primers used in this study.

**Primers for quantitative real-time RT-qPCR**	
**Name**	**Sequence**
SYP121-F	5'- GGTGGTGTGAACCTCGACAA -3'
SYP121-R	5'- TCCAACGTCACCGTCCATTT -3'
WDL4-F	5'- TGATGCAACAGGAGGTGAAG -3'
WDL4-R	5'- GCTTTGGCTGAGACTTTGTTATT -3'
WDL5-F	5'- CCAGACTTCACATCCCACAGA -3'
WDL5-R	5'- CGGCTCTGTCTTCATTCATCC -3'
PIN1-F	5'- TCGTCGCTCTCTTCGCCGTT -3'
PIN1-R	5'- TGCGGCTGAGTTTGCACCAG -3'
VAMP721-F	5'- GCACGGTGATCCTCGTTGAG -3'
VAMP721-R	5'- CCGTCGCAGTTGTAGGTGAA -3'
IAA1-F	5'- ACTAGAACTTTCTTGCGTCAGA -3'
IAA1-R	5'- GATTTGTGTTTTTGCAGGAGGA -3'
GFP-F	5'- GGACGACGGCAACTACAAGA -3'
GFP-R	5'- CTCGATGTTGTGGCGGATCT -3'
cLUC-F	5'- TCATCGTTGACCGCCTGAAG -3'
cLUC-R	5'- ATGTTGGGGTGTTGGAGCAA -3'
nLUC-F	5'- TGATTACACCCGAGGGGGAT -3'
nLUC-R	5'- CCAGATCCACAACCTTCGCT -3'
EF1α-F	5'- GACATGAGGCAGACTGTTGCA -3'
EF1α-R	5'- CCGGTTGGGTCCTTCTTGT -3'
18S rRNA-F	5'- CGGCTACCACATCCAAGGAA -3'
18S rRNA-R	5'- GCTGGAATTACCGCGCGGCT -3'

**Primers for genotyping**	
**Name**	**Sequence**
syp121-5-LP	5'- ACCATTGCATGTCCTTTTCAC -3'
syp121-5-RP	5'- ACTTGTCGAGGTTCACACCAC -3'
wdl4-1-LP	5'- CTCCAACCTGAACGAAAATCC -3'

wdl4-1-RP	5'- TTTGGAAGTTGTCCCTGAATG -3'
LBb1	5'- GCGTGGACCGCTTGCTGCAACT -3'
wdl4-2-LP	5'- ATGGCGTCCGAGGATTTGAATA -3'
wdl4-2-RP	5'- CCTGAAGCTGCATCCTTAGAAAT -3'
syp121-7-F	5'- GTGGAAATCAGTCGATCTAGGG -3'
syp121-7-R	5'- CTTCCTCTTCCTTGTTCTTG -3'

**Primers for cloning**		
**Name**	**Sequence**	**Plasmids**
WDL4-EcoRI-F<br>*WDL4*-SmaI-R	5'- GAATTCATGGCGTCCGAGGATTTGAA -3'<br>5'- CCCGGGGCCTCCCACTACAACCTCAC TCG -3'	pGADT7
WDL4-SalI-F<br>*WDL4*-KpnI-R	5'-GTCGACATATGGCGTCCGAGGATTTGAA TATTG -3'<br>5'- GGTACCTCAGCCTCCCACTACAACCTCAC TCG -3'	pCAMBIA1300 (mCherry)
WDL4-XbaI-F<br>*WDL4*-SalI-R	5'- TCTAGAATGGCGTCCGAGGATTTG -3'<br>5'- GTCGACTCAGCCTCCCACTACAACCTCAC -3'	pMALc2x
WDL4-KpnI-F<br>*WDL4*-SalI-R	5'- GGTACCATGGCGTCCGAGGATTTGAATA TTG -3'<br>5'- GTCGACGCCTCCCACTACAACCTCAC -3'	35S: nLUC
WDL4-PstI-F<br>*WDL4*-SalI-R	5'- CTGCAGATGGCGTCCGAGGATTTG -3'<br>5'- GTCGACGCCTCCCACTACAACC -3'	pCAMBIA1390 (*UBQ10: CFP*)
WDL4N-SalI-F<br>*WDL4N*-SmaI-R	5'- GTCGACATGGCGTCCGAGGATTTGAATA TTG -3'<br>5'- CCCGGGCTTGCTTGCCATTTCAGCTTTC -3'	pCAMBIA1390 (*WDL4pro: GFP*)
WDL4C-SalI-F<br>*WDL4C*-SmaI-R	5'- GTCGACATGGACGACGAAGACACTCGTT -3'<br>5'- CCCGGGGCCTCCCACTACAACCTCACTCG -3'	pCAMBIA1390 (*WDL4pro: GFP*)
WDL4N-XbaI-F<br>*WDL4N*-SalI-R	5'- TCTAGAATGGCGTCCGAGGATTTGAATA TTG -3'<br>5'- GTCGACCTTGCTTGCCATTTCAGCTTTC -3'	35S: GFP

WDL4C-XbaI-F<br>*WDL4C*-SalI-R	5’- TCTAGAATGGACGACGAAGACACTCGTT -3’<br>5’- GTCGACGCCTCCCACTACAACCTCACTCG -3’	35S: GFP
WDL4N-EcoRI-F<br>*WDL4N*-SmaI-R	5’- GAATTCATGGCGTCCGAGGATTTGAATATTG -3’<br>5’- CCCGGGCTTGCTTGCCATTTCAGCTTTC -3’	pGADT7
WDL4C-EcoRI-F<br>*WDL4C*-SmaI-R	5’- GAATTCATGGACGACGAAGACACTCGTT -3’<br>5’- CCCGGGGCCTCCCACTACAACCTCACTCG -3’	pGADT7
WDL4N-SpeI-F<br>*WDL4N*-KpnI-R	5’- ACTAGTATGGCGTCCGAGGATTTGAAT -3’<br>5’- GGTACCCTTGCTTGCCATTTCAGCTTT -3’	pCAMBIA1300 (GFP)
WDL4C-SpeI-F<br>*WDL4C*-KpnI-R	5’- ACTAGTATGGACGACGAAGACACT -3’<br>5’- GGTACCGCCTCCCACTACAACCTC -3’	pCAMBIA1300 (GFP)
SYP121pro-PstI-F<br>*SYP121pro*-SalI-R	5’- CTGCAGGTTGTATATTTTTTGTTTGTTTGAG -3’<br>5’- GTCGACTTAAAATTCGAAAAATAGAG -3’	pCAMBIA1390 (GFP)
SYP121-BamHI-F<br>*SYP121*-EcoRI-R	5’- GGATCCATGAACGATTTGTTTTCCAGCTC -3’<br>5’- GAATTCTCAACGCAATAGACGCCTTG -3’	pCAMBIA1390 (GFP)
SYP121-SalI-F<br>*SYP121*-SpeI-R	5’- GTCGACATATGAACGATTTGTTTTCCAGCTCATTC -3’<br>5’- ACTAGTTCAACGCAATAGACGCCTTGCCTGA -3’	pCAMBIA1300 (mCherry)
SYP121-BamHI-F<br>*SYP121*-SalI-R	5’- GGATCCAATGAACGATTTGTTTTCCAGCTCATTC -3’<br>5’- GTCGACACGCAATAGACGCCTTGCCT -3’	35S: cLUC
SYP121-SalI-F<br>*SYP121*-SpeI-R	5’- GTCGACATATGAACGATTTGTTTTCCAGCTCATTC -3’<br>5’- ACTAGTTCAACGCAATAGACGCCTTGCCTGA -3’	35S: nLUC
SYP121ΔN-KpnI-F	5’- GGTACCATGGGTGGTGTGAACCTCGAC -3’	35S: cLUC

SYP121ΔN-BamHI-R	5’- GGATCCACGCAATAGACGCCTTGC -3’	
SYP121ΔC-EcoRV-F	5’- GATATCATGAACGATTTGTTTTCCAGCTC -3’	pET30a (+) (His-GFP)
SYP121ΔC-BamHI-R	5’- GGATCCACATGTCCATTTTCGCGTGTTCTT -3’	
SYP121ΔC-EcoRI-F	5’- GAATTCATGAACGATTTGTTTTCCAG -3’	pGBKT7
SYP121ΔC-BamHI-R	5’- GGATCCACATGTCCATTTTCGCGTG -3’	
SYP121N-EcoRI-F	5’- GAATTCATGAACGATTTGTTTTCCAG -3’	pGBKT7
SYP121N-BamHI-R	5’- GGATCCGGTTGATCCCGCGGGATTC -3’	
SYP121HQ-EcoRI-F	5’- GAATTCATGGGTGGTGTGAACCTCGAC -3’	pGBKT7
SYP121HQ-BamHI-R	5’- GGATCCACATGTCCATTTTCGCGTG -3’	
SYP121ΔC-EcoRI-F	5’- GAATTCATGAACGATTTGTTTTCCAG -3’	pGADT7
SYP121ΔC-BamHI-R	5’- GGATCCACATGTCCATTTTCGCGTG -3’	
VAMP721ΔC-EcoRI-F	5’- GAATTCATGGCGCAACAATCGTTGATC -3’	
VAMP721ΔC-BamHI-R	5’- GGATCCTCTTCTCATCTGCGTTCCTG -3’	pGBKT7
VAMP721-KpnI-F	5’- GGTACCATGGCGCAACAATCGTTGATC -3’	35S: cLUC
VAMP721-SalI-R	5’- GTCGACTTAACACTTAAACCCATGGC -3’	
KEULE-BamHI-F	5’- GGATCCATGTCGTACTCTGACTCCGA -3’	pET30a (+)
KEULE-SalI-R	5’- GTCGACTATTTGGAGATCGTCTAAAG -3’	
WDL5-BamHI-F	5’- GGATCCATGGACCCTGAGAGTATCATG -3’	

WDL5-SalI-R	5’- GTCGACTTAATGCTCAACAGCAACCGCTTC -3’	pMALc2x
WDL5-SalI-F<br>*WDL5*-KpnI-R	5’- GTCGACATATGGACCCTGAGAGTATCATG -3’<br>5’- GGTACCTTAATGCTCAACAGCAACCGCTTC -3’	pCAMBIA1300 (mCherry)
SYP132ΔC-EcoRI-F<br>*SYP132ΔC*-SalI-R	5’-ATGGCCATGGAGGCCGAATTCATGAACGATCTTCTGAAGGGTTCG -3’<br>5’- ATGCGGCCGCTGCAGGTCGACTCCCGATTGCACGTGATCTAC -3’	pGBKT7
VAMP714ΔC -EcoRI-F<br>*VAMP714ΔC*-SalI-R	5’-TGGCCATGGAGGCCGAATTCATGGCGATTGTCTATGCTGT -3’<br>5’- TGCGGCCGCTGCAGGTCGACCTTAGCATTTTTCATCCAAA -3’	pGBKT7

Primers for CRISPR/CAS9 (*SYP121*)

**Name**	**Sequence**	**Plasmids**
DT1-BsF	5’- ATATATGGTCTCGATTGATCCCGCGGGATCAACCGGGTT -3’	pCBC-DT1T2
DT1-F0	5’- TGATCCCGCGGGATCAACCGGGTTTTAGAGCTAGAAATAGC -3’	pHEC401
DT2-R0	5’- AACTCCATTTTAGACCGGAGATCAATCTCTTAGTCGACTCTAC -3’	
DT2-BsR:	5’- ATTATTGGTCTCGAAACTCCATTTTAGACCGGAGATC -3’	
U626-IDF:	5’- TGTCCCAGGATTAGAATGATTAGGC -3’	
U629-IDF:	5’- TTAATCCAAACTACTGCAGCCTGAC -3’	
U629-IDR:	5’- AGCCCTCTTCTTTCGATCCATCAAC -3’	
